# Diagnostic accuracy of deep learning using speech samples in depression: a systematic review and meta-analysis

**DOI:** 10.1093/jamia/ocae189

**Published:** 2024-07-16

**Authors:** Lidan Liu, Lu Liu, Hatem A Wafa, Florence Tydeman, Wanqing Xie, Yanzhong Wang

**Affiliations:** Department of Population Health Sciences, School of Life Course and Population Sciences, Faculty of Life Sciences & Medicine, King's College London, London, SE1 1UL, United Kingdom; Department of Population Health Sciences, School of Life Course and Population Sciences, Faculty of Life Sciences & Medicine, King's College London, London, SE1 1UL, United Kingdom; Department of Population Health Sciences, School of Life Course and Population Sciences, Faculty of Life Sciences & Medicine, King's College London, London, SE1 1UL, United Kingdom; Department of Population Health Sciences, School of Life Course and Population Sciences, Faculty of Life Sciences & Medicine, King's College London, London, SE1 1UL, United Kingdom; Department of Intelligent Medical Engineering, School of Biomedical Engineering, Anhui Medical University, Hefei, 230032, China; Department of Psychology, School of Mental Health and Psychological Sciences, Anhui Medical University, Hefei, 230032, China; Beth Israel Deaconess Medical Center, Harvard Medical School, Harvard University, Boston, MA, 02115, United States; Department of Population Health Sciences, School of Life Course and Population Sciences, Faculty of Life Sciences & Medicine, King's College London, London, SE1 1UL, United Kingdom

**Keywords:** depression, deep learning, speech, meta-analysis, systematic review

## Abstract

**Objective:**

This study aims to conduct a systematic review and meta-analysis of the diagnostic accuracy of deep learning (DL) using speech samples in depression.

**Materials and Methods:**

This review included studies reporting diagnostic results of DL algorithms in depression using speech data, published from inception to January 31, 2024, on PubMed, Medline, Embase, PsycINFO, Scopus, IEEE, and Web of Science databases. Pooled accuracy, sensitivity, and specificity were obtained by random-effect models. The diagnostic Precision Study Quality Assessment Tool (QUADAS-2) was used to assess the risk of bias.

**Results:**

A total of 25 studies met the inclusion criteria and 8 of them were used in the meta-analysis. The pooled estimates of accuracy, specificity, and sensitivity for depression detection models were 0.87 (95% CI, 0.81-0.93), 0.85 (95% CI, 0.78-0.91), and 0.82 (95% CI, 0.71-0.94), respectively. When stratified by model structure, the highest pooled diagnostic accuracy was 0.89 (95% CI, 0.81-0.97) in the handcrafted group.

**Discussion:**

To our knowledge, our study is the first meta-analysis on the diagnostic performance of DL for depression detection from speech samples. All studies included in the meta-analysis used convolutional neural network (CNN) models, posing problems in deciphering the performance of other DL algorithms. The handcrafted model performed better than the end-to-end model in speech depression detection.

**Conclusions:**

The application of DL in speech provided a useful tool for depression detection. CNN models with handcrafted acoustic features could help to improve the diagnostic performance.

**Protocol registration:**

The study protocol was registered on PROSPERO (CRD42023423603).

## Background and objective

Depression disorder is a common mental disorder, involving a low mood, loss of interest in everyday life, and other symptoms, which lead to burden, disability, and even suicide.^1^ World Health Organization reports that 280 million people were diagnosed with depression in 2019, including almost 10% of children and adolescents.^2^ Early recognition of depression reduces the complication of treatment, shortens the course of the disease, and provides positive treatment outcomes.^3^

Currently, clinical symptoms, supplemented with objective physiological indicators and questionnaires, are considered to diagnose depression. Clinical symptoms must last for 2 weeks at least to confirm a diagnosis of depression, leaving patients with limited care or treatment during the early stage of the disorder.^4^ Moreover, subjective factors, such as patients’ expressions, cultures, and attitudes, may make the diagnosis of depression more complex with a greater probability of misdiagnosis. Therefore, recent studies suggest using signal processing methods, including audio,^5^ videos,^6^ and electroencephalogram (EEG),^7^ to increase the diagnostic accuracy of depression.

Speech has been proven as an important biomarker for depression detection since people with depression turn out to speak at a lower rate, give more prolonged pauses, and change less pitch than normal people.^8^^,^^9^ Compared with other biomarkers, such as videos, EEG, and skin conductance, speech has many advantages. First, it is easy and non-invasive to collect using smartphones or computers. Second, it contains various information related to depression symptoms and this information is difficult to hide. Third, it reduces privacy exposure for patients.^5^ A neural network is a series of connected weighted nodes that models the biological nervous system function of the human brain.^10^ Neural networks provide effective tools in speech processing since they have the ability to automatically learn available features from raw speech, reducing the subjectivity in manual feature selection.^11^ The successful applications of DL algorithms in speech signal processing and classification present a novel opportunity to improve the performance of automatic depression detection.^12^

Recent reviews addressed various psychiatric disorders and artificial techniques, and they gave a comprehensive explanation of the importance of applying artificial intelligence to support clinical diagnosis.^5^^,^^11^^,^^13^^,^^14^ However, to the best of our knowledge, few reviews focused on the use of deep learning (DL) algorithms to detect depression in speech till now. Thus, we aim to provide a systematic review and meta-analysis to evaluate the diagnostic performance of the DL algorithms in detecting and classifying depression using speech samples.

## Methods

This review was conducted according to Preferred Reporting Items for a Systematic Review and Meta-analysis of Diagnostic Test Accuracy Studies statement.^15^^,^^16^ The study protocol was registered on PROSPERO (CRD42023423603).

### Search strategy

We searched the following datasets: PubMed, Medline, Embase, PsycINFO, Scopus, IEEE, and Web of Science databases up to January 31, 2024, using the following keywords including, but not limited to, combinations of the following: depressi*, depressive disorder*, deep learning, machine learning, artificial intelligence, neural network, automat*, sound, speech, voice, acoustic*, audio, vowel, vocal, pitch, prosody. The complete search strategy is presented in the Supplementary Material.

### Inclusion and exclusion criteria

This review includes studies evaluating the diagnostic accuracies of DL algorithms in depression using speech samples. After screening, we excluded the studies published with no full text.

We also excluded studies: (1) developed multimodal methods to detect depression; (2) without reporting original data (eg, review or protocol studies without applying DL algorithms); (3) without reporting diagnostic test results [true positive (TP), false positive (FP), true negative (TN), false negative (FN)]; (4) without using DL algorithms.

### Study selection and data extraction

Titles and abstracts of the retrieved literature were screened for eligibility. Relevant articles were read in full, and data were extracted from the articles that met all inclusion criteria. Two authors (L.L. and L.L.) conducted all these steps individually, and a third researcher (Y.W.) was included to solve the disagreements and uncertainties in the study selection process and data extraction process by discussion.

The following data was extracted from the included studies independently: title, authors, year of publication, diagnosis standard (scales), features, classification methods, model structure, and diagnostic test results (TP, TN, FP, and FN).

### Statistical analysis

Sensitivity, specificity, and accuracy were calculated with a 95% CI based on the TP, TN, FP, and FN values that were extracted from the included studies for meta-analysis. The accuracy can be calculated as: Accuracy = (TP + TN) / (TP + TN + FP + FN). The interpretation of accuracy could be affected by the prevalence of depression because, in cases of very high or very low prevalence, accuracy might not provide a complete picture of a test’s performance. In our included studies, the prevalence is neither too high nor too low. Therefore, our pooled estimates of accuracy, especially their interpretation, are unlikely to be affected by the varying prevalence of the condition. We used the ${\;I}^{2}$ to measure the heterogeneity across studies and subgroups, with 25%, 50%, and 75% being considered as thresholds to indicate the low, moderate, and high heterogeneity, respectively.^17^ A *P*-value was used to measure the statistic, and *P* < .05 was considered statistically significant. A funnel plot was used to assess publication bias. All the analyses were performed using RStudio version 12.0 with the meta package.^18^

Pooled estimates of depression detection in speech using DL algorithms were obtained. Leave-one-out method and subgroup analysis were used to evaluate the sensitivity and reduce the heterogeneity among the studies. SROC curve which represents the performance of a diagnostic test was also built to describe the relationship between test sensitivity and specificity.^19^

### Assessment of bias

QUADAS-2 recommended by the Cochrane Collaboration was used to evaluate the risk of bias in each study by two authors (L.L. and L.L.), and the uncertainties were discussed with a third researcher (Y.W.). QUADAS-2 evaluates 4 key domains including patient selection, index test, reference standard, and flow and timing. Each domain is analyzed in terms of risk of bias, with particular attention given to concerns about applicability in the first three domains. The assessment of bias was conducted using the Review Manager Software version 5.3.^20^

## Results

### Literature search

**Figure 1.**
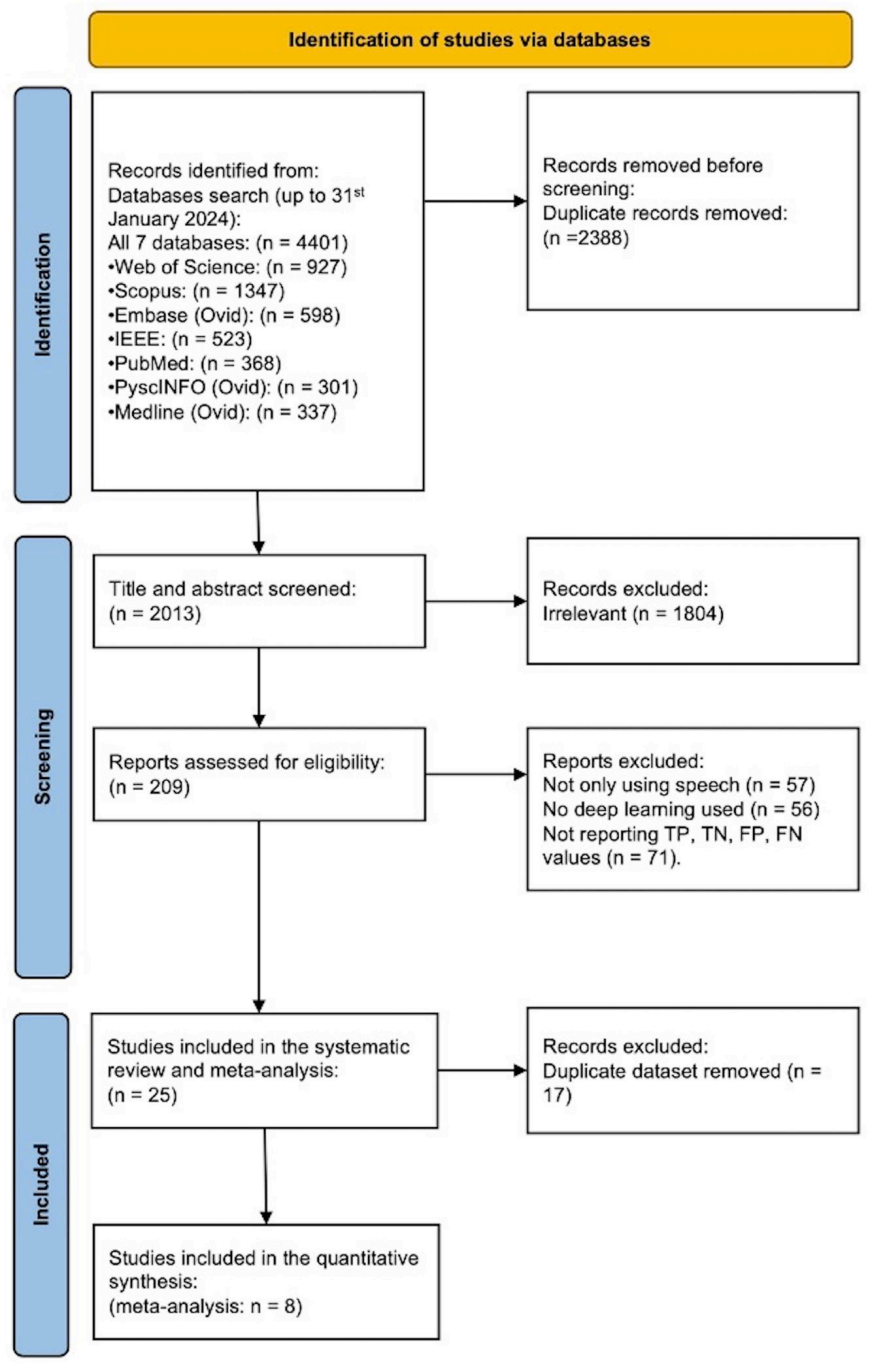
PRISMA flowchart. Study selection for systematic review and meta-analysis.

A total of 2013 records were selected after duplicate removal. After screening the title and abstract, 1804 articles were excluded, with 209 articles being assessed for full-text review. Of these, 56 articles investigated speech signal processing in detecting depression but were excluded because they did not use DL algorithms. Another 57 articles excluded because not only did they apply speech samples but also other formats of data, including texts, sentiments, images, videos, and EEG. Finally, 71 articles were excluded since they did not report TP, TN, FP, and FN values used in the meta-analysis, and the remaining 25 articles were included in the systematic review. Some of them used the same dataset to explore the performance of different DL models, so we selected the studies with the highest accuracy score of each dataset to do the meta-analysis, and 8 studies were included (Figure 1). Of all included studies, the Distress Analysis Interview Corpus-Wizard-of-Oz (DAIC-WOZ) set is the most-used (*n* = 16) dataset, and we used these studies to do the qualitative analysis from a technical perspective.^21^ An upward trend for publications was shown in the past 3 years. 20 papers (80%) were published since 2022, and all eligible papers were published after 2019. Table 1 summarizes the main characteristics of all eligible papers, the ones in bold font were selected for the meta-analysis.

**Table 1.** Characteristics of the included studies.

Study	Datasets	Scales	Languages	Features extracted	Model structure	DL models	Prevalence (case/total sample size)	Sens.	Spec.	Acc.
Chlasta et al. (2019)^22^	DAIC-WOZ	PHQ-8	English	spectrogram	End-to-End	CNN (ResNet-50)	28% (30/107)	0.14	1.00	0.78
Saidi et al. (2020)^23^	DAIC-WOZ	PHQ-8	English	spectrogram	End-to-End	CNN(CNN-SVM)	31% (38/122)	0.71	0.65	0.68
A. Othmani et al. (2021)^24^	DAIC-WOZ	PHQ-8	English	spectrogram, MFCC	Handcrafted	CNN (EmoAudioNet)	/(/182)	0.46	0.84	0.73
Rejaibi et al. (2022)^25^	DAIC-WOZ	PHQ-8	English	MFCC	Handcrafted	LSTM	30% (54/182)	0.35	0.59	0.54
Homsiang et al. (2022)^26^	DAIC-WOZ	PHQ-8	English	spectrogram	End-to-End	CNN	50% (35/70)	1.00	0.90	0.95
Sardari et al. (2022)^27^	DAIC-WOZ	PHQ-8	English	raw audio	End-to-End	CNN	30% (49/161)	0.68	0.72	0.70
Ravi et al. (2022)^28^	DAIC-WOZ	PHQ-8	English	LLDs	Handcrafted	DepAudioNet	30% (42/142)	0.77	0.67	0.74
Cui et al. (2022)^29^	DAIC-WOZ	PHQ-8	English	MFCC	Handcrafted	CNN (Federated Learning)	30% (56/189)	0.82	0.94	0.91
Zhou et al. (2022)^30^	DAIC-WOZ	PHQ-8	English	LLDs, MFCC, functionals, spectrogram	Handcrafted	CNN	30% (56/189)	0.83	0.83	0.83
Yin et al. (2023)^31^	DAIC-WOZ	PHQ-8	English	MFCC	Handcrafted	Transformer-CNN-CNN	47% (42/89)	0.92	0.95	0.94
Tian et al. (2023)^32^	DAIC-WOZ	PHQ-8	English	fusion features	Handcrafted	CNN	30% (56/189)	0.86	0.88	0.88
Feng et al. (2023)^33^	DAIC-WOZ	PHQ-8	English	acoustic features	Handcrafted	CNN	30% (42/142)	0.58	0.87	0.77
**Ishimaru et al. (2023)^34^**	**DAIC-WOZ**	**PHQ-8**	**English**	**65-dimensional feature vectors**	**Handcrafted**	**GCNN (Graphical CNN)**	**30% (56/189)**	**0.95**	**0.98**	**0.97**
Marriwala et al. (2023)^35^	DAIC-WOZ	PHQ-8	English	spectrogram	End-to-End	CNN	30% (56/189)	0.63	0.42	0.57
Bhavya et al. (2023)^36^	DAIC-WOZ	PHQ-8	English	MFCC	Handcrafted	LSTM	30% (56/189)	0.79	0.50	0.70
Ravi et al. (2024)^37^	DAIC-WOZ	PHQ-8	English	acoustic features	Handcrafted	CNN (CNN-LSTM)	30% (42/142)	0.67	0.91	0.83
**Gupta et al. (2023)^38^**	**SH2-FS**	**PHQ-9**	**English**	**fusion features**	**Handcrafted**	**CNN (DAttn_Conv 2D LSTM)**	**17% (97/566)**	**0.99**	**0.96**	**0.99**
**Suparatpinyo et al. (2023)^39^**	**Thai corpus**	**PHQ-9**	**Thai**	**spectrogram**	**End-to-End**	**CNN (ResNet-50)**	**68% (63/93)**	**0.74**	**0.80**	**0.77**
**Yang et al. (2023)^40^**	**NRAC**	**PHQ-9**	**Mandarin**	**spectrogram**	**End-to-End**	**CNN (DALF)**	**76% (108/142)**	**0.91**	**0.76**	**0.87**
Jenei et al. (2020)^41^	Hungarian	BDI-II	Hungarian	MFCC, formant frequency	Handcrafted	CNN	50% (91/182)	0.80	0.88	0.84
**Jenei et al. (2021)^42^**	**Hungarian**	**BDI-II**	**Hungarian**	**fusion features**	**Handcrafted**	**CNN**	**50% (91/182)**	**0.88**	**0.83**	**0.85**
**Wang et al. (2022)^43^**	**Chinese Hospital Dataset**	**HAMD**	**Mandarin**	**deep spectrum features**	**End-to-End**	**CNN (Multi-mlp)**	**48% (76/157)**	**0.88**	**0.81**	**0.84**
**Wang et al. (2022)^44^**	**Northwest Normal University**	**BDI**	**Mandarin**	**MFCC**	**Handcrafted**	**CNN (ResNet-18)**	**50% (25/50)**	**0.76**	**0.74**	**0.75**
Du et al. (2023)^45^	MODMA	PHQ-9	Mandarin	LPC, MFCC	Handcrafted	CNN (CNN-LSTM)	44% (23/52)	0.90	0.82	0.86
**Das et al. (2024)^46^**	**MODMA**	**PHQ-9**	**Mandarin**	**MFCC, spectrogram**	**Handcrafted**	**CNN (CNN-ANN)**	**44% (23/52)**	**0.94**	**0.86**	**0.9**

Notes: Acc., Accuracy; Sens., Sensitivity; Spec., Specificity. The studies included in the meta-analysis are shown in bold font.

### Characteristics of the included studies

#### Datasets and languages

Several speech depression datasets were used to train models, and the speeches were generally recorded during the diagnosis conversation between clinicians and participants. DAIC-WOZ, which is a part of Distress Analysis Interview Corpus developed in 2016 Audio–Visual Emotion Challenge (AVEC), is the most commonly used dataset in speech depression detection.^47^ Besides, the Multi-modal Open Dataset for Mental-disorder Analysis (MODMA),^48^ Hungarian Depression Speech Database,^42^ Sonde Health Free Speech (SH2-FS),^49^ three Mandarin datasets,^40^^,^^43^^,^^44^ and one Thai dataset recruited by researchers were also used in the included studies.^39^ Some studies used more than 1 dataset to test the performance of their proposed model. Among all included studies, 17 studies (68%) used English datasets, 5 studies (20%) used Mandarin datasets, 2 studies (8%) used Hungarian datasets, and only one used Thai dataset.

#### Diagnostic scales

In speech depression datasets, diagnostic scale scores are set as training labels. PHQ-8 scores of each participant were recorded in DAIC‐WOZ, and the score of 10 was set as the threshold to decide whether the participant was diagnosed with depression or not.^50^ Besides, other questionnaires, such as the Hamilton Rating Scale for Depression (HAMD),^51^ Beck Depression Inventory-II (BDI-II),^52^ and PHQ-9,^53^ were also used as the assessments to detect depression.

### Method assessment within the DAIC-WOZ dataset

#### Speech processing

**Figure 2.**
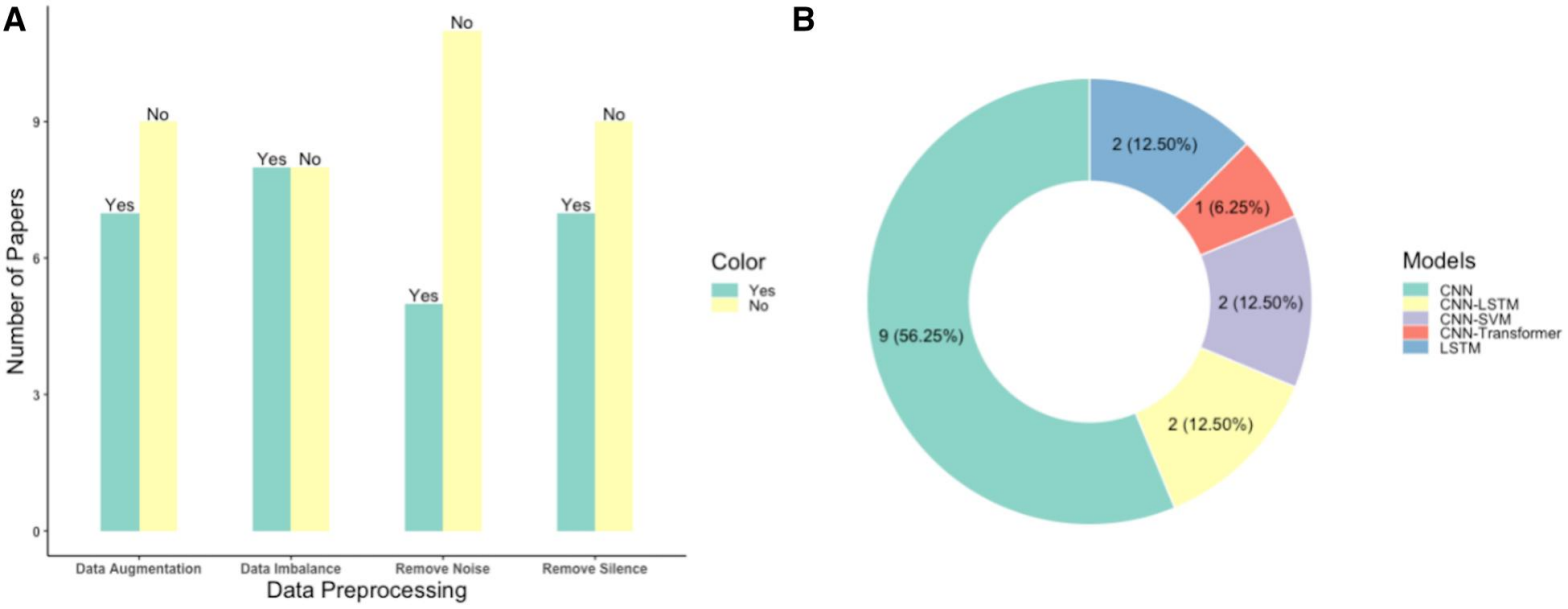
Speech preprocessing and deep learning models. (A) The number of studies that used preprocessing steps, such as removing silence. (B) The number of studies that used different types of DL models.

In the development of an automatic speech recognition system, preprocessing is considered the first phase to train a robust and efficient model.^54^ Fourteen studies (87.5%) mentioned at least one speech preprocessing procedure with 50% of the papers applying various methods to tackle data imbalance as shown in Figure 2A. This result is unsurprising, given that the DAIC-WOZ dataset consists of 146 depressed subjects and only 43 healthy participants, highlighting the critical issue of data imbalance in achieving good performance.

Speech segments of varying lengths are used as inputs to the model, enriching the dataset and accommodating DL models (Figure S12A). Yin and colleagues segmented the speech into 9-second fragments, achieving the highest performance across all studies with 0.94 in accuracy and 0.92 in sensitivity.^31^ Moreover, fragments over 10 seconds in length exhibited the highest specificity (Table 2). In Figure S12B, we can find that all studies employed either a train-test split or a train-validation-test split to prevent overfitting to the training data and accurately assess the model’s performance.

**Table 2.** Performance comparison of characteristics of the studies using the DAIC-WOZ dataset.

	Acc. (mean)	Sens. (mean)	Spec. (mean)	References
**Speech segmentation**				
Less than 1 s (*n* = 3)	0.76	0.62	0.81	^33^ ^,^ ^34^^,^ ^25^
Less than 5 s (*n* = 4)	0.74	0.71	0.74	^28^ ^,^ ^37^^,^ ^23^^,^ ^27^
Less than 10 s (*n* = 1)	**0.94**	**0.92**	0.95	^31^
No less than 10 s (*n* = 2)	0.84	0.48	**0.97**	^22^ ^,^ ^29^
NA (*n* = 6)	0.78	0.76	0.73	^36^ ^,^ ^26^^,^ ^35^^,^ ^24^^,^ ^32^^,^ ^42^
**Features**				
Spectrograms (*n* = 5)	0.75	0.61	0.77	^22^ ^,^ ^33^^,^ ^26^^,^ ^35^^,^ ^23^
Fusion features (*n* = 4)	0.74	0.62	0.78	^24^ ^,^ ^25^^,^ ^32^^,^ ^30^
LLDs (*n* = 3)	**0.85**	0.79	**0.85**	^34^ ^,^ ^28^^,^ ^37^
MFCCs (*n* = 3)	**0.85**	**0.84**	0.80	^36^ ^,^ ^29^^,^ ^31^
Raw audio (*n* = 1)	0.70	0.68	0.72	^27^
**Models**				
CNN (*n* = 9)	0.82	0.70	0.85	^22^ ^,^ ^29^^,^ ^33^^,^ ^26^^,^ ^34^^,^ ^35^^,^ ^24^^,^ ^32^^,^ ^30^
CNN-LSTM (*n* = 2)	0.79	0.72	0.79	^28^ ^,^ ^37^
CNN-SVM (*n* = 2)	0.69	0.70	0.69	^23^ ^,^ ^27^
LSTM (*n* = 2)	0.62	0.57	0.55	^36^ ^,^ ^25^
CNN-transformer (*n* = 1)	**0.94**	**0.92**	**0.95**	^31^
**Neural network layers**				
No more than 5 (*n* = 10)	**0.79**	0.75	0.79	^36^ ^,^ ^29^^,^ ^33^^,^ ^26^^,^ ^28^^,^ ^37^^,^ ^25^^,^ ^23^^,^ ^32^^,^ ^31^
No more than 10 (*n* = 3)	0.76	0.68	0.74	^34^ ^,^ ^35^^,^ ^24^
No more than 30 (*n* = 2)	0.77	**0.76**	0.78	^27^ ^,^ ^30^
More than 30 (*n* = 1)	0.78	0.14	**1.00**	^22^
**Loss functions**				
Cross Entropy (*n* = 5)	**0.84**	**0.70**	**0.91**	^29^ ^,^ ^33^^,^ ^34^^,^ ^24^^,^ ^37^
Mean Squared Error (*n* = 2)	0.62	0.51	0.66	^25^ ^,^ ^27^
**Optimizers**				
ADAM (*n* = 7)	0.77	0.67	0.78	^33^ ^,^ ^34^^,^ ^35^^,^ ^24^^,^ ^25^^,^ ^31^^,^ ^30^
SGD (*n* = 2)	**0.84**	0.48	**0.97**	^22^ ^,^ ^29^
AdaDelta (*n* = 1)	0.70	**0.68**	0.72	^27^

Notes: Acc., Accuracy; Sens., Sensitivity; Spec., Specificity. The best performance for each characteristic of the studies is shown in bold font.

Feature engineering is one of the most crucial steps of traditional machine learning based speech depression detection research and the main purpose of many studies considered in this review is to avoid this step by developing DL for automatic feature learning.^26^^,^^27^^,^^35^^,^^55^ Based on the results shown in Table 2, it is evident that LLDs and MFCCs-based models achieved over 80% accuracy, surpassing other types of features. Besides, 3 included studies compared the performance of speech depression detection with multimodal depression detection, and acoustic features.^24^^,^^25^^,^^35^ All these 3 studies present that using multimodal features enhances the performance of speech depression detection models.

#### Deep learning methodology

Compared with clinical diagnosis, DL algorithms can learn high-level features automatically. In this review, we divided the DL models used in the included studies into the following groups: convolutional neural network (CNN), CNN-long short-term memory (LSTM), CNN-support vector machine (SVM), LSTM, and CNN-Transformer. Figure 2B shows CNN is the most commonly used DL algorithm, with 56.25% of the studies using it directly as the depression detection model. Additionally, 25% of studies employed CNN as a feature extraction or dimension reduction method, followed by the use of LSTM (12.5%) or SVM (12.5%) as a classifier for depression detection. The CNN-Transformer architecture shows the highest performance among all studies (Table 2), which indicates that the transformer holds promising potential for depression detection using speech data.^31^

**Figure 3.**
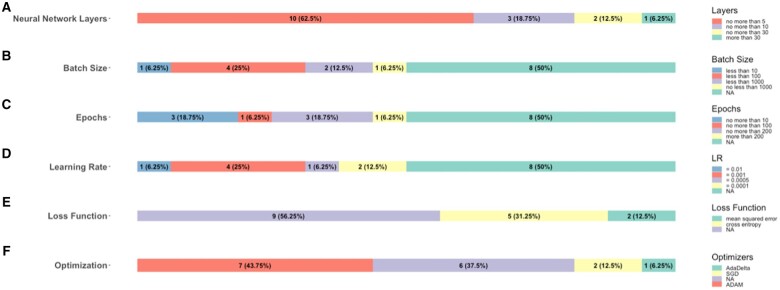
Hyperparameters choices. (A) Distribution of the number of neural network layers. (B) The number of studies that used different batch sizes. (C) Distribution of the number of epochs. (D) The number of studies that used different learning rates. (E) The number of studies that used different loss functions. (F) The number of studies that used different optimizers.

In Figure 3, we present a visualization of the distribution of hyperparameters in DL models. As shown in Figure 3, 50% of studies did not report batch size, 50% of studies did not report epochs, 50% of studies did not report learning rate, 56.25% of studies did not report loss functions and 43.75% of studies did not report optimizers. These hyperparameters may affect the model’s performance to some extent, highlighting the importance of selecting appropriate hyperparameters. The number of neural network layers does not necessarily exceed 5 in most studies (62.5%) under consideration (Figure 3A), and such kind of studies achieved the highest accuracy, which is 0.79 (Table 2). Cross-entropy was the most commonly used (31.25%) loss function, outperforming mean square error in terms of accuracy (0.84), sensitivity (0.70), and specificity (0.91) (Figure 3E and Table 2).

#### Evaluation measures

**Figure 4.**
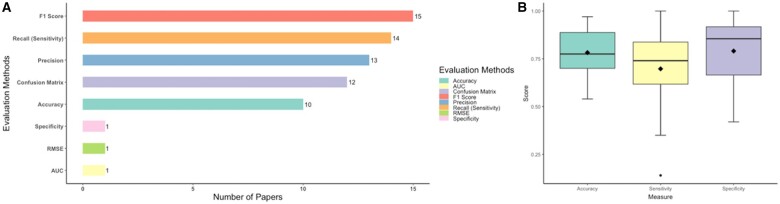
Model performance evaluation. (A) the number of studies that used different evaluation methods. (B) The boxplot across studies of accuracy, sensitivity, and specificity.

The types of performance metrics used by the included studies focusing on speech depression detection are shown in Figure 4A. Most studies (over 80%) used F1-score, accuracy, recall, and precision which were derived from confusion matrices to evaluate the performance of the DL models, but these metrics were not commonly used by clinicians in evaluating diagnostic tests. Instead, sensitivity (recall), specificity and ROC AUC, which are also derived from diagnostic test results, are clinically relevant and commonly used performance measures for diagnostic tests. Based on Figure 4B, we found that the included studies achieved good results in terms of accuracy and specificity (over 75%), but slightly lagged in sensitivity.

#### Comparison between deep learning and machine learning

Four studies compared their proposed methods with machine learning methods.^23^^,^^27^^,^^31^^,^^36^ SVM is the most commonly used machine learning algorithm, and 3 studies compared their proposed methods with SVM.^23^^,^^27^^,^^36^ In all these 4 studies, the proposed DL methods performed better than machine learning methods.

#### Summary

The papers reviewed displayed varying degrees of speech depression detection. (1) Data preprocessing: segmentation of speech varied in length across papers, with notable performance achieved through longer segments (more than 5 s). (2) Features: the preference for DL models suggests a shift away from traditional feature engineering, with promising results observed particularly with LLDs and MFCCs. (3) Models: CNN emerges as the predominant choice among DL architectures for depression detection, with CNN-Transformer demonstrating the highest performance. While hyperparameters significantly impact model performance, many studies lack specificity in their selection, underscoring the importance of fine-tuning for optimal results. (4) Evaluation: Overall, the models using the DAIC-WOZ dataset generally achieved good accuracy and specificity (over 75%), and the sensitivity lagged slightly.

### Diagnostic accuracy of deep learning in depression detection

**Figure 5.**
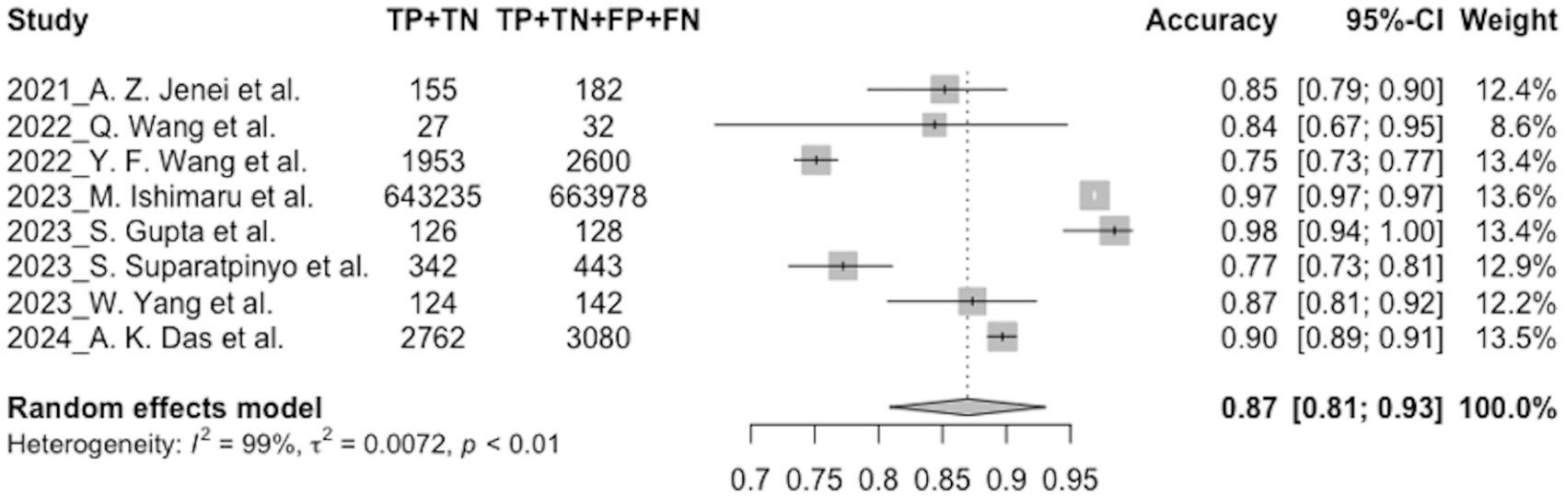
Forest plot for the pooled accuracy.

Overall, 8 studies with 670 585 preprocessed speech samples in the test sets were included in the meta-analysis, and all these studies were published in the last 5 years (2021-2024). Our study reports the evaluation parameters of accuracy, sensitivity, and specificity. The pooled estimate of classification accuracy for depression detection models was 0.87 (95% CI, 0.81-0.93, $I^{2}$ = 99%). Meta-analysis showed the pooled estimate to be specific (0.85, 95% CI, 0.78-0.91, $I^{2}$ = 99%), but with a lower sensitivity (0.82, 95% CI, 0.71-0.94, $I^{2}$ = 100%). The random-effect model was used due to the high heterogeneity in the meta-analysis. Figure 5 represents the accuracy forest plots of all included studies. The forest plots of the pooled sensitivity and specificity can be found in supplementary files (Figures S1 and S2). SROC curve for the test is also shown in supplementary files (Figure S3).

### Sensitivity analysis

**Figure 6.**
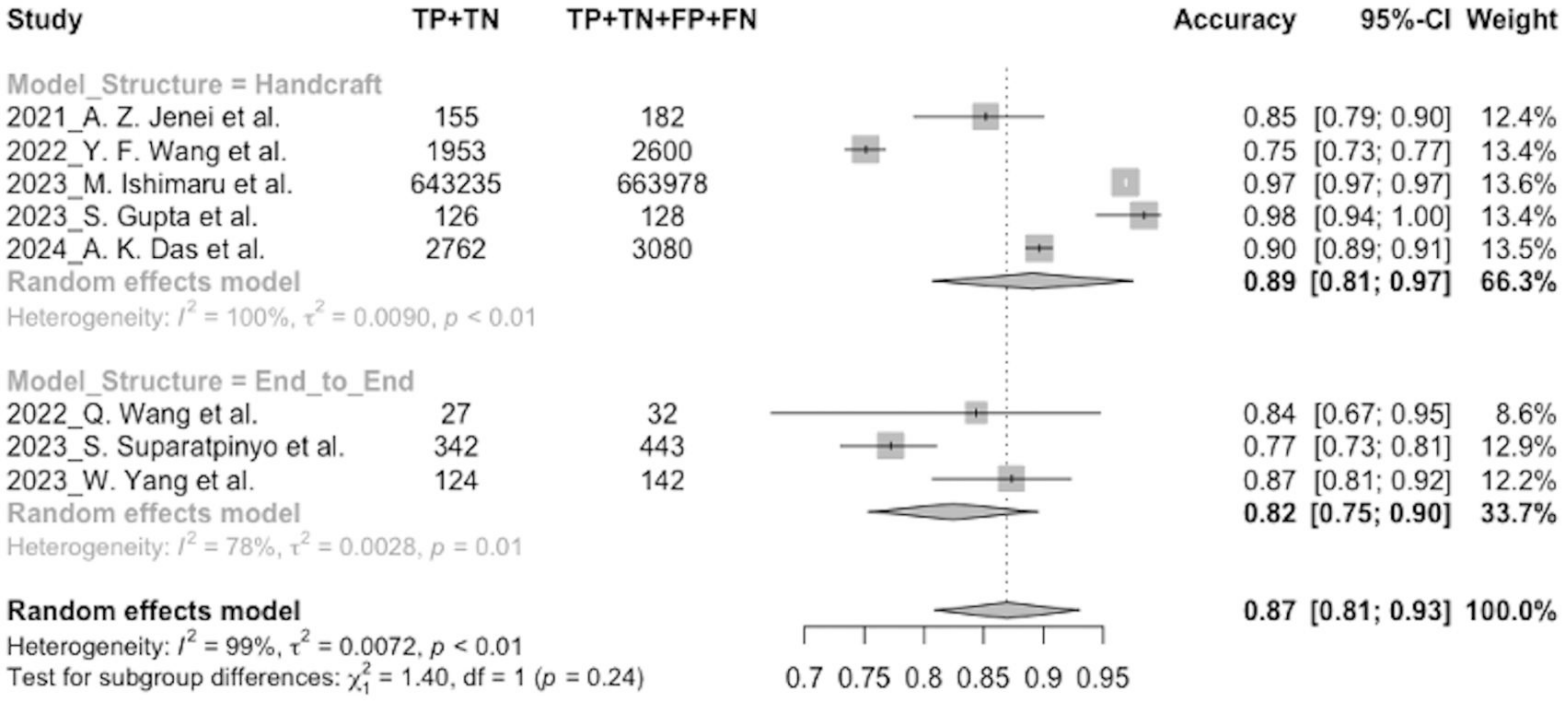
Forest plot of the pooled accuracy for the model structure subgroup.

Considering the high heterogeneity, subgroup analysis was undertaken to excavate the potential factors. $I^{2}$ dropped significantly in specificity (from 100% to 0%), accuracy (from 100% to 78%), and sensitivity (from 100% to 88%) of the end-to-end group in the model structure subgroup. In this situation, the accuracy and the specificity of the handcrafted group (accuracy: 0.89, 95% CI, 0.81-0.97, $I^{2}$ = 100%; specificity: 0.87, 95% CI, 0.78-0.96, $I^{2}$ = 99%) was higher than the end-to-end group (accuracy: 0.82, 95% CI, 0.75-0.90, $I^{2}$ = 78%; specificity: 0.80, 95% CI, 0.75-0.85, $I^{2}$ = 0%), but the sensitivity of the end-to-end group (0.84, 95% CI, 0.73-0.95, $I^{2}$ = 88%) was higher than the handcrafted group (0.81, 95% CI, 0.64-0.99, $I^{2}$ = 100%). The forest plot of the pooled accuracy for the model structure subgroup is shown in Figure 6, and the other forest plots for the pooled sensitivity and specificity for the model structure subgroup can be found in supplementary files (Figures S4 and S5).

Since speech samples in some included studies were segmented from audios, the sample size in one study (*n* = 663 978) was extremely larger than the others. Leave-one-out test was conducted to minimize the influence of the particular study.^34^ While omitting each study, the pooled estimates of accuracy (0.85-0.89), sensitivity (0.80-0.87), and specificity (0.82-0.87) changed a little. The plots for the leave-one-out results of the pooled accuracy, sensitivity, and specificity can be found in supplementary files (Figures S6-S8).

### Quality assessment

QUADAS-2 was used to rate the overall methodological quality in our study, and the Figures S9 and S10 present the plots illustrating the risk of bias and applicability concerns. The included studies achieved an average score of 3.3 out of 4 in the risk of bias section, and 3.1 out of 4 in the applicability concerns section, thereby affirming the high quality of the studies. The funnel plot (Figure S11) was slightly asymmetric, indicating modest publication bias in all the included studies. Specifically, the shape of the plot suggests that smaller data sizes with low accuracy were less likely to get published.

## Discussion

### Summary of key findings

To our knowledge, our study is the first review on the diagnostic performance of DL for depression detection from speech samples providing both systematic review (narrative summary of 25 studies) and meta-analysis (quantitative assessment of a subset of 8 studies). We found that across all included studies, the pooled estimate of the accuracy for depression detection was 0.87, and the specificity (0.85) was higher than the sensitivity (0.82). The handcrafted model obtained better evaluation results (accuracy: 0.89) in the subgroup analysis than the end-to-end model (accuracy: 0.82).

#### Speech features for depression detection

A recent review found that a set of bio-acoustic features, including source, spectral, prosodic, and formants, could improve the classification performance for depression detection.^56^ In addition, Zhao et al reported that acoustic characteristics were associated with the severity of depressive symptoms and might be objective biomarkers of depression.^57^ The findings are consistent with the present study that handcrafted model structure gave better performance than end-to-end model structure. This is because the handcrafted model structure contains various kinds of selected acoustic information, such as source and formants. Besides, our results showed acoustic features were promising, reliable, and objective biomarkers to support depression diagnosis using DL.

#### Superior performance of deep learning in depression detection

A recent systematic review (but not meta-analysis) suggested that SVM was the most popular classifier used among all machine learning (ML) methods in depression detection.^58^ Bhadra and his colleagues merged DL techniques into a single classifier group to compare with other ML algorithms owing to the limited studies accessible, which gave a comprehensive description of all ML algorithms but remained extensible for further research on DL.^58^ In the present review, some included studies confirmed that DL surpasses previous ML methods for automated diagnosis of depression, such as SVM, Random Forest, and Gradient Boosting Tree.^27^^,^^36^^,^^40^, As mentioned in the present review, the prevailing emphasis lies on CNN models, and it may be beneficial to explore more DL methods in depression detection. Although DL has less interpretability than other computational methods, it has shown great potential to assist in the diagnosis of depression.

#### Deep learning model structure strategies

Wu and colleagues summarized in their systematic survey that applying DL in depression detection could be built in two structures: (1) extract hand-craft acoustic features, and then implement classification methods; (2) put raw audio or spectrograms into an end-to-end DL architecture to do both feature extraction and classification by itself.^13^ To explore the performance of these two structures, we applied the subgroup analysis of the model structure in the meta-analysis. The pooled estimates of depression detection performance in the handcrafted structure were higher than the end-to-end structure, which provided evidence that the good performance of DL might rely on the strategies of model structures. Since its lack of interpretability, it is still limited to applying the end-to-end deep model to solve real-world clinical problems.

#### Future development of deep learning

Applying DL algorithms on speech samples to support clinical diagnosis for depression disorders was novel, but still needs further development. First, the performance of the automatic speech depression detection models may be influenced by different languages, cultures, and environments. Gabor Kiss and his colleagues found that training the models with Hungarian speech samples while testing them with German speech samples gave a worse performance than training and testing the models with only Hungarian speech samples.^59–61^ Second, due to the difficulties and privacy issues of collecting depression speeches, issues of small sample size and data imbalance need to be solved before training a DL model. Third, the outperformance of CNN related model may be partly explained by the common interest in CNN, since most studies included in the systematic review focused on optimizing parameters for the CNN-related algorithms. Therefore, the performance of other DL algorithms remains to be deciphered. Fourth, the explainability of DL models is a limitation in speech depression detection. It is difficult to understand how decisions are made by DL, which is crucial for gaining trust and acceptance in clinical settings.

### Clinical and research implication

The increasing prevalence of depression is a significant burden that could overwhelm mental health services capacity. Although automated depression detection allows wide screening of a larger population and ameliorate the increasing demand placed on health services, these techniques should still be used as supplementing methods to detect early signs of depression. Despite the positive attitudes of clinicians toward diagnosis-supported techniques, rolling out such novel applications on a wider scale remains challenging until knowledge of DL is obtained and experience is acquired in using those techniques in the diagnosis of depression.^62^ Therefore, future research should better involve physicians to improve the feasibility of techniques and require clinical trials to further explore the utility of diagnosis-supported tools. Besides, since speech is easy to collect using smartphones, future research can focus on implementing remote monitors on smartphones to obtain valuable information from real-time response and relapse, support physicians’ decisions, and generate immediate diagnosis feedback.

### Source of heterogeneity

The pooled results in the meta-analysis represented significant heterogeneity among the studies. There may be many reasons, including the various sample sizes based on speech segmentation, different speech languages and cultures, and different methodologies. In this study, we analyzed subgroup and leave-one-out results to explore the sources of heterogeneity. $I^{2}$ dropped significantly in specificity when dividing studies based on model structure (from 100% to 0%), which indicated that model structure might be the major cause of heterogeneity. Besides, heterogeneity was slightly lower in specificity when omitting the study with the biggest sample size,^34^ providing evidence that the speech segmentation methods and the speech sample sizes also influenced the heterogeneity.

### Limitations

Our study has several limitations. First, only a limited number of studies were included in the systematic review because most studies did not report the original TP, TN, FP, and FN scores, and this may lead to underpowered pooled estimates. An updated meta-analysis could be performed in the future when source studies are sufficient to make the results more robust. Second, most studies included used the same dataset, so we selected the best performance model from each dataset to ensure the validity and reliability of the meta-analysis. The limited number of studies in the meta-analysis made it difficult to stratify the studies into different subgroups to explore the source of heterogeneity. Third, we did not do the meta-analysis based on AUROC scores which were usually used to describe the performance of classification models since only 3 studies reported AUROC scores in all included studies.^29^^,^^41^^,^^44^

## Conclusions

We conducted a comprehensive systematic review and meta-analysis reporting the application of DL algorithms in speech to detect depression. The review confirms that using DL in speech to support the clinical diagnosis of depression is a promising method with excellent performance. CNN model with handcrafted acoustic features training on an appropriately balanced dataset was shown to be the best method in depression detection. Further studies could focus on multi-lingual and cross-lingual speech depression detection, DL algorithms exploration and optimization, and multimodal features combination. In addition, researchers should report diagnostic evaluation measures, such as sensitivity and specificity, to interpret DL results in real-world clinical settings.

## Supplementary Material

ocae189_Supplementary_Data

## Data Availability

The data underlying this article are available in the article and in its online supplementary material.

## References

[1] Lepine J-P. Epidemiology, burden, and disability in depression and anxiety. J Clin Psychiatry. 2001;62(Suppl 13):4-10. 11434418

[2] World Health Organization. Mental Disorders. Accessed August 2, 2023. https://www.who.int/news-room/fact-sheets/detail/mental-disorders

[3] Lecrubier Y. The burden of depression and anxiety in general medicine. J Clin Psychiatry. 2021;62(Suppl 8):4-9. 12108821

[4] Guha M. Diagnostic and statistical manual of mental disorders: DSM-5. Ref Rev. 2014;28(3):36-37.

[5] Low DM , Bentley KH, Ghosh SS. Automated assessment of psychiatric disorders using speech: a systematic review. Laryngoscope Investig Otolaryngol. 2020;5(1):96-116. 10.1002/lio2.354 PMC7042657 32128436

[6] Pampouchidou A , Pediaditis M, Kazantzaki E, et al Automated facial video-based recognition of depression and anxiety symptom severity: cross-corpus validation. Mach Vis Appl. 2020;31(4):30.

[7] Khosla A , Khandnor P, Chand T. Automated diagnosis of depression from EEG signals using traditional and deep learning approaches: a comparative analysis. Biocybern Biomed Eng. 2022;42(1):108-142.

[8] Wu Z , Fang Y. Comorbidity of depressive and anxiety disorders: challenges in diagnosis and assessment. Shanghai Arch Psychiatry. 2014;26(4):227-231. 25317009 10.3969/j.issn.1002-0829.2014.04.006 PMC4194005

[9] Koops S , Brederoo SG, Boer JN, et al Speech as a biomarker for depression. CNS Neurol Disord Drug Targets. 2023;22(2):152-160. 34961469 10.2174/1871527320666211213125847

[10] Abiodun OI , Jantan A, Omolara AE, et al State-of-the-art in artificial neural network applications: a survey. Heliyon. 2018;4(11):e00938. 30519653 10.1016/j.heliyon.2018.e00938 PMC6260436

[11] Mehrish A , Majumder N, Bharadwaj R, et al A review of deep learning techniques for speech processing. Inf Fusion. 2023;99:101869.

[12] Cummins N , Baird A, Schuller BW. Speech analysis for health: Current state-of-the-art and the increasing impact of deep learning. Methods. 2018;151:41-54. 30099083 10.1016/j.ymeth.2018.07.007

[13] Wu P , Wang R, Lin H, et al Automatic depression recognition by intelligent speech signal processing: a systematic survey. CAAI Trans Intel Tech. 2022;8(3):701-711.

[14] Cummins N , Scherer S, Krajewski J, et al A review of depression and suicide risk assessment using speech analysis. Speech Commun. 2015;71:10-49.

[15] Page MJ , McKenzie JE, Bossuyt PM, et al The PRISMA 2020 statement: an updated guideline for reporting systematic reviews. BMJ. 2021;372(8284):n71. 33782057 10.1136/bmj.n71 PMC8005924

[16] McInnes MDF , Moher D, Thombs BD, et al Preferred reporting items for a systematic review and meta-analysis of diagnostic test accuracy studies: the PRISMA-DTA statement. JAMA. 2018;319(4):388-396. 29362800 10.1001/jama.2017.19163

[17] Higgins JP , Green S. Guide to the contents of a cochrane protocol and review. Cochrane Handbook for Systematic Reviews of Interventions 2008;51–79.

[18] Schwarzer G. meta: an R package for meta-analysis. R News. 2007;7(3):40-45.

[19] Moses LE , Shapiro D, Littenberg B. Combining independent studies of a diagnostic test into a summary ROC curve: data‐analytic approaches and some additional considerations. Stat Med. 1993;12(14):1293-1316. 8210827 10.1002/sim.4780121403

[20] Whiting PF , Rutjes AWS, Westwood ME, et al QUADAS-2: A revised tool for the quality assessment of diagnostic accuracy studies. Ann Intern Med. 2011;155(8):529-536. 22007046 10.7326/0003-4819-155-8-201110180-00009

[21] Gratch J , Artstein R, Lucas G, et al The distress analysis interview corpus of human and computer interviews. *Proceedings of the 9th International Conference on Language Resources and Evaluation*, LREC. European Language Resources Association; 2014:3123-3128.

[22] Chlasta K , Wołk K, Krejtz I. Automated speech-based screening of depression using deep convolutional neural networks. Procedia Comput Sci. 2019;164:618-628.

[23] Saidi A , Othman SB, Saoud SB. Hybrid CNN-SVM classifier for efficient depression detection system. *2020 4th International Conference on Advanced Systems and Emergent Technologies (IC_ASET)*. IEEE; 2020:229-234.

[24] Othmani A , Kadoch D, Bentounes K, et al Towards robust deep neural networks for affect and depression recognition from speech. *ICPR International Workshops and Challenges*. Springer Link; 2021:5-19.

[25] Rejaibi E , Komaty A, Meriaudeau F, et al MFCC-based recurrent neural network for automatic clinical depression recognition and assessment from speech. Biomed Signal Process Control. 2022;71:103107.

[26] Homsiang P , Treebupachatsakul T, Kiatrungrit K, et al Classification of depression audio data by deep learning. *2022 14th Biomedical Engineering International Conference (BMEiCON)*. IEEE; 2022:1-4.

[27] Sardari S , Nakisa B, Rastgoo MN, et al Audio based depression detection using convolutional autoencoder. Expert Syst Appl. 2022;189:116076.

[28] Ravi V , Wang J, Flint J, et al A step towards preserving speakers’ identity while detecting depression via speaker disentanglement. Interspeech. 2022;2022:3338-3342. 36341467 10.21437/interspeech.2022-10798 PMC9635494

[29] Cui Y , Li Z, Liu L, et al Privacy-preserving speech-based depression diagnosis via federated learning. *2022 44th Annual International Conference of the IEEE Engineering in Medicine & Biology Society (EMBC)*. IEEE; 2022:1371-1374. 10.1109/EMBC48229.2022.9871861 36085955

[30] Zhou Z , Guo Y, Hao S, et al Hierarchical multifeature fusion via audio-response-level modeling for depression detection. IEEE Trans Comput Soc Syst. 2023;10(5):2797-2805.

[31] Yin F , Du J, Xu X, et al Depression detection in speech using transformer and parallel convolutional neural networks. Electronics. 2023;12(2):328.

[32] Tian H , Zhu Z, Jing X. Deep learning for depression recognition from speech. Mobile Netw Appl. 2023;28:1-16.

[33] Feng K , Chaspari T. A knowledge-driven vowel-based approach of depression classification from speech using data augmentation. *ICASSP 2023—2023 IEEE International Conference on Acoustics, Speech and Signal Processing (ICASSP)*. IEEE; 2023:1-5.

[34] Ishimaru M , Okada Y, Uchiyama R, et al Classification of depression and its severity based on multiple audio features using a graphical convolutional neural network. Int J Environ Res Public Health. 2023;20(2):1588. 36674342 10.3390/ijerph20021588 PMC9864471

[35] Vandana MN , Chaudhary D. A hybrid model for depression detection using deep learning. Meas: Sensors. 2023;25:100587.

[36] B S , Nayak DS, Dmello RC, et al Machine learning applied to speech emotion analysis for depression recognition. *2023 International Conference for Advancement in Technology (ICONAT)*. IEEE; 2023:1-5.

[37] Ravi V , Wang J, Flint J, et al Enhancing accuracy and privacy in speech-based depression detection through speaker disentanglement. Comput Speech Lang. 2024;86:101605. 38313320 10.1016/j.csl.2023.101605 PMC10836190

[38] Gupta S , Agarwal G, Agarwal S, et al Depression detection using cascaded attention based deep learning framework using speech data. Multimedia Tools Appl. 2024;83(4):66135-66173.

[39] Suparatpinyo S , Soonthornphisaj N. Smart voice recognition based on deep learning for depression diagnosis. Artif Life Robot. 2023;28(2):332-342.

[40] Yang W , Liu J, Cao P, et al Attention guided learnable time-domain filterbanks for speech depression detection. Neural Netw. 2023;165:135-149. 37285730 10.1016/j.neunet.2023.05.041

[41] Jenei AZ , Kiss G. Possibilities of recognizing depression with convolutional networks applied in correlation structure. *2020 43rd International Conference on Telecommunications and Signal Processing (TSP)*. IEEE; 2020:101-4.

[42] Jenei AZ , Kiss G. Severity estimation of depression using convolutional neural network. Periodica Polytechnica Electr Eng Comput Sci. 2021;65(3):227-234.

[43] Wang Q , Liu N. Speech detection of depression based on multi-mlp. *IEEE International Conference on Bioinformatics and Biomedicine (BIBM).* IEEE; 2022:3896-3898.

[44] Wang Y , Lu X, Shi D. MFCC-based deep convolutional neural network for audio depression recognition. *2022 International Conference on Asian Language Processing (IALP)*. IEEE; 2022:162-166.

[45] Du M , Liu S, Wang T, et al Depression recognition using a proposed speech chain model fusing speech production and perception features. J Affect Disord. 2023;323:299-308. 36462607 10.1016/j.jad.2022.11.060

[46] Das AK , Naskar R. A deep learning model for depression detection based on MFCC and CNN generated spectrogram features. Biomed Signal Process Control. 2024;90:105898.

[47] Valstar M , Gratch J, Schuller B, et al AVEC 2016: depression, mood, and emotion recognition workshop and challenge. *Proceedings of the 6th International Workshop on Audio/Visual Emotion Challenge*. ACM Multimedia; 2016:3-10.

[48] Cai H , Yuan Z, Gao Y, et al A multi-modal open dataset for mental-disorder analysis. Sci Data. 2022;9(1):178. 35440583 10.1038/s41597-022-01211-x PMC9018722

[49] Huang Z , Epps J, Joachim D. Exploiting vocal tract coordination using dilated CNNS for depression detection in naturalistic environments. *ICASSP 2020—2020 IEEE International Conference on Acoustics, Speech and Signal Processing*. IEEE; 2020:6549-6553.

[50] Kroenke K , Strine TW, Spitzer RL, et al The PHQ-8 as a measure of current depression in the general population. J Affect Disord. 2009;114(1-3):163-173. 18752852 10.1016/j.jad.2008.06.026

[51] Hamilton M. A rating scale for depression. J Neurol Neurosurg Psychiatry. 1960;23(1):56-62. 14399272 10.1136/jnnp.23.1.56 PMC495331

[52] Beck AT , Steer RA, Ball R, et al Comparison of beck depression inventories-IA and-II in psychiatric outpatients. J Pers Assess. 1996;67(3):588-597. 8991972 10.1207/s15327752jpa6703_13

[53] Kroenke K , Spitzer RL, Williams JBW. The PHQ-9. J Gen Intern Med. 2001;16(9):606-613. 11556941 10.1046/j.1525-1497.2001.016009606.x PMC1495268

[54] Ibrahim YA , Odiketa JC, Ibiyemi TS. Preprocessing technique in automatic speech recognition for human computer interaction: an overview. Ann Comput Sci Ser. 2017;15(1):186-191.

[55] Liu Z , Hu B, Yan L, et al Detection of depression in speech. *2015 International Conference on Affective Computing and Intelligent Interaction (ACII)*. IEEE; 2015:743-747.

[56] Almaghrabi SA , Clark SR, Baumert M. Bio-acoustic features of depression: a review. Biomed Signal Process Control. 2023;85:105020.

[57] Zhao Q , Fan H-Z, Li Y-L, et al Vocal acoustic features as potential biomarkers for identifying/diagnosing depression: a cross-sectional study. Front Psychiatry. 2022;13:815678. 10.3389/fpsyt.2022.815678 35573349 PMC9095973

[58] Bhadra S , Kumar CJ. An insight into diagnosis of depression using machine learning techniques: a systematic review. Curr Med Res Opin. 2022;38(5):749-771. 35129401 10.1080/03007995.2022.2038487

[59] Laukka P , Neiberg D, Elfenbein HA. Evidence for cultural dialects in vocal emotion expression: acoustic classification within and across five nations. Emotion. 2014;14(3):445-449. 24749633 10.1037/a0036048

[60] Kiss G , Tulics MG, Sztahó D, et al Language independent detection possibilities of depression by speech. Recent Adv Nonlinear Speech Process. 2016;48:103-114.

[61] Albouy P , Mehr SA, Hoyer RS, et al Spectro-temporal acoustical markers differentiate speech from song across cultures. Nat Commun. 2024;15(1):4835. 10.1038/s41467-024-49040-3 PMC11156671 38844457

[62] Kerst A , Zielasek J, Gaebel W. Smartphone applications for depression: a systematic literature review and a survey of health care professionals’ attitudes towards their use in clinical practice. European Arch Psychiatry Clin Neurosci. 2020;270(2):139-152. 30607530 10.1007/s00406-018-0974-3

