# Supplementary material for: Diagnostic accuracy of deep learning using speech samples in depression: a systematic review and meta-analysis
Source: J Am Med Inform Assoc. 2024 Jul 16;31(10):2394–404. doi: 10.1093/jamia/ocae189 (PMC11413444; doi:10.1093/jamia/ocae189)
Supplement: ocae189_Supplementary_Data [file ocae189_supplementary_data.zip › ocae189_Supplementary_Data/Search Strategy.docx]

**Search Strategy**

**Embase (Ovid)**

1. depressi*.mp. [mp=title, abstract, heading word, drug trade name, original title, device manufacturer, drug manufacturer, device trade name, keyword heading word, floating subheading word, candidate term word]
2. depressive disorder*.mp. [mp=title, abstract, heading word, drug trade name, original title, device manufacturer, drug manufacturer, device trade name, keyword heading word, floating subheading word, candidate term word]
3. deep learning.mp. [mp=title, abstract, heading word, drug trade name, original title, device manufacturer, drug manufacturer, device trade name, keyword heading word, floating subheading word, candidate term word]
4. machine learning.mp. [mp=title, abstract, heading word, drug trade name, original title, device manufacturer, drug manufacturer, device trade name, keyword heading word, floating subheading word, candidate term word]
5. Artificial Intelligence.mp. [mp=title, abstract, heading word, drug trade name, original title, device manufacturer, drug manufacturer, device trade name, keyword heading word, floating subheading word, candidate term word]
6. neural network.mp. [mp=title, abstract, heading word, drug trade name, original title, device manufacturer, drug manufacturer, device trade name, keyword heading word, floating subheading word, candidate term word]
7. automat*.mp. [mp=title, abstract, heading word, drug trade name, original title, device manufacturer, drug manufacturer, device trade name, keyword heading word, floating subheading word, candidate term word]
8. sound.mp. [mp=title, abstract, heading word, drug trade name, original title, device manufacturer, drug manufacturer, device trade name, keyword heading word, floating subheading word, candidate term word]
9. speech.mp. [mp=title, abstract, heading word, drug trade name, original title, device manufacturer, drug manufacturer, device trade name, keyword heading word, floating subheading word, candidate term word]
10. voice.mp. [mp=title, abstract, heading word, drug trade name, original title, device manufacturer, drug manufacturer, device trade name, keyword heading word, floating subheading word, candidate term word]
11. acoustic*.mp. [mp=title, abstract, heading word, drug trade name, original title, device manufacturer, drug manufacturer, device trade name, keyword heading word, floating subheading word, candidate term word]
12. audio.mp. [mp=title, abstract, heading word, drug trade name, original title, device manufacturer, drug manufacturer, device trade name, keyword heading word, floating subheading word, candidate term word]
13. vowel.mp. [mp=title, abstract, heading word, drug trade name, original title, device manufacturer, drug manufacturer, device trade name, keyword heading word, floating subheading word, candidate term word]
14. vocal.mp. [mp=title, abstract, heading word, drug trade name, original title, device manufacturer, drug manufacturer, device trade name, keyword heading word, floating subheading word, candidate term word]
15. pitch.mp. [mp=title, abstract, heading word, drug trade name, original title, device manufacturer, drug manufacturer, device trade name, keyword heading word, floating subheading word, candidate term word]
16. prosody.mp. [mp=title, abstract, heading word, drug trade name, original title, device manufacturer, drug manufacturer, device trade name, keyword heading word, floating subheading word, candidate term word]
17. 3 OR 4 OR 5 OR 6 OR 7
18. 8 OR 9 OR 10 OR 11 OR 12 OR 13 OR 14 OR 15
19. 1 OR 2
20. 17 AND 18 AND 19

**Medline (Ovid)**

1. depressi*.mp. [mp=title, book title, abstract, original title, name of substance word, subject heading word, floating sub-heading word, keyword heading word, organism supplementary concept word, protocol supplementary concept word, rare disease supplementary concept word, unique identifier, synonyms, population supplementary concept word, anatomy supplementary concept word]
2. depressive disorder*.mp. [mp=title, book title, abstract, original title, name of substance word, subject heading word, floating sub-heading word, keyword heading word, organism supplementary concept word, protocol supplementary concept word, rare disease supplementary concept word, unique identifier, synonyms, population supplementary concept word, anatomy supplementary concept word]
3. deep learning.mp. [mp=title, book title, abstract, original title, name of substance word, subject heading word, floating sub-heading word, keyword heading word, organism supplementary concept word, protocol supplementary concept word, rare disease supplementary concept word, unique identifier, synonyms, population supplementary concept word, anatomy supplementary concept word]
4. machine learning.mp. [mp=title, book title, abstract, original title, name of substance word, subject heading word, floating sub-heading word, keyword heading word, organism supplementary concept word, protocol supplementary concept word, rare disease supplementary concept word, unique identifier, synonyms, population supplementary concept word, anatomy supplementary concept word]
5. Artificial Intelligence.mp. [mp=title, book title, abstract, original title, name of substance word, subject heading word, floating sub-heading word, keyword heading word, organism supplementary concept word, protocol supplementary concept word, rare disease supplementary concept word, unique identifier, synonyms, population supplementary concept word, anatomy supplementary concept word]
6. neural network.mp. [mp=title, book title, abstract, original title, name of substance word, subject heading word, floating sub-heading word, keyword heading word, organism supplementary concept word, protocol supplementary concept word, rare disease supplementary concept word, unique identifier, synonyms, population supplementary concept word, anatomy supplementary concept word]
7. automat*.mp. [mp=title, book title, abstract, original title, name of substance word, subject heading word, floating sub-heading word, keyword heading word, organism supplementary concept word, protocol supplementary concept word, rare disease supplementary concept word, unique identifier, synonyms, population supplementary concept word, anatomy supplementary concept word]
8. sound.mp. [mp=title, book title, abstract, original title, name of substance word, subject heading word, floating sub-heading word, keyword heading word, organism supplementary concept word, protocol supplementary concept word, rare disease supplementary concept word, unique identifier, synonyms, population supplementary concept word, anatomy supplementary concept word]
9. speech.mp. [mp=title, book title, abstract, original title, name of substance word, subject heading word, floating sub-heading word, keyword heading word, organism supplementary concept word, protocol supplementary concept word, rare disease supplementary concept word, unique identifier, synonyms, population supplementary concept word, anatomy supplementary concept word]
10. voice.mp. [mp=title, book title, abstract, original title, name of substance word, subject heading word, floating sub-heading word, keyword heading word, organism supplementary concept word, protocol supplementary concept word, rare disease supplementary concept word, unique identifier, synonyms, population supplementary concept word, anatomy supplementary concept word]
11. acoustic*.mp. [mp=title, book title, abstract, original title, name of substance word, subject heading word, floating sub-heading word, keyword heading word, organism supplementary concept word, protocol supplementary concept word, rare disease supplementary concept word, unique identifier, synonyms, population supplementary concept word, anatomy supplementary concept word]
12. audio.mp. [mp=title, book title, abstract, original title, name of substance word, subject heading word, floating sub-heading word, keyword heading word, organism supplementary concept word, protocol supplementary concept word, rare disease supplementary concept word, unique identifier, synonyms, population supplementary concept word, anatomy supplementary concept word]
13. vowel.mp. [mp=title, book title, abstract, original title, name of substance word, subject heading word, floating sub-heading word, keyword heading word, organism supplementary concept word, protocol supplementary concept word, rare disease supplementary concept word, unique identifier, synonyms, population supplementary concept word, anatomy supplementary concept word]
14. vocal.mp. [mp=title, book title, abstract, original title, name of substance word, subject heading word, floating sub-heading word, keyword heading word, organism supplementary concept word, protocol supplementary concept word, rare disease supplementary concept word, unique identifier, synonyms, population supplementary concept word, anatomy supplementary concept word]
15. pitch.mp. [mp=title, book title, abstract, original title, name of substance word, subject heading word, floating sub-heading word, keyword heading word, organism supplementary concept word, protocol supplementary concept word, rare disease supplementary concept word, unique identifier, synonyms, population supplementary concept word, anatomy supplementary concept word]
16. prosody.mp. [mp=title, book title, abstract, original title, name of substance word, subject heading word, floating sub-heading word, keyword heading word, organism supplementary concept word, protocol supplementary concept word, rare disease supplementary concept word, unique identifier, synonyms, population supplementary concept word, anatomy supplementary concept word]
17. 3 OR 4 OR 5 OR 6 OR 7
18. 8 OR 9 OR 10 OR 11 OR 12 OR 13 OR 14 OR 15
19. 1 OR 2
20. 17 AND 18 AND 19

**Psyinfo (Ovid)**

1. depressi*.mp. [mp=title, abstract, heading word, table of contents, key concepts, original title, tests & measures, mesh word]
2. depressive disorder*.mp. [mp=title, abstract, heading word, table of contents, key concepts, original title, tests & measures, mesh word]
3. deep learning.mp. [mp=title, abstract, heading word, table of contents, key concepts, original title, tests & measures, mesh word]
4. machine learning.mp. [mp=title, abstract, heading word, table of contents, key concepts, original title, tests & measures, mesh word]
5. Artificial Intelligence.mp. [mp=title, abstract, heading word, table of contents, key concepts, original title, tests & measures, mesh word]
6. neural network.mp. [mp=title, abstract, heading word, table of contents, key concepts, original title, tests & measures, mesh word]
7. automat*.mp. [mp=title, abstract, heading word, table of contents, key concepts, original title, tests & measures, mesh word]
8. sound.mp. [mp=title, abstract, heading word, table of contents, key concepts, original title, tests & measures, mesh word]
9. speech.mp. [mp=title, abstract, heading word, table of contents, key concepts, original title, tests & measures, mesh word]
10. voice.mp. [mp=title, abstract, heading word, table of contents, key concepts, original title, tests & measures, mesh word]
11. acoustic*.mp. [mp=title, abstract, heading word, table of contents, key concepts, original title, tests & measures, mesh word]
12. audio.mp. [mp=title, abstract, heading word, table of contents, key concepts, original title, tests & measures, mesh word]
13. vowel.mp. [mp=title, abstract, heading word, table of contents, key concepts, original title, tests & measures, mesh word]
14. vocal.mp. [mp=title, abstract, heading word, table of contents, key concepts, original title, tests & measures, mesh word]
15. pitch.mp. [mp=title, abstract, heading word, table of contents, key concepts, original title, tests & measures, mesh word]
16. prosody.mp. [mp=title, abstract, heading word, table of contents, key concepts, original title, tests & measures, mesh word]
17. 3 OR 4 OR 5 OR 6 OR 7
18. 8 OR 9 OR 10 OR 11 OR 12 OR 13 OR 14 OR 15
19. 1 OR 2
20. 17 AND 18 AND 19

**PubMed**

1. #1 "depressi*"[All Fields] OR (("depressed"[All Fields] OR "depression"[MeSH Terms] OR "depression"[All Fields] OR "depressions"[All Fields] OR "depression s"[All Fields] OR "depressive disorder"[MeSH Terms] OR ("depressive"[All Fields] AND "disorder"[All Fields]) OR "depressive disorder"[All Fields] OR "depressivity"[All Fields] OR "depressive"[All Fields] OR "depressively"[All Fields] OR "depressiveness"[All Fields] OR "depressives"[All Fields]) AND "disorder*"[All Fields])
2. #2 "deep learning"[MeSH Terms] OR ("deep"[All Fields] AND "learning"[All Fields]) OR "deep learning"[All Fields] OR ("machine learning"[MeSH Terms] OR ("machine"[All Fields] AND "learning"[All Fields]) OR "machine learning"[All Fields]) OR ("artificial intelligence"[MeSH Terms] OR ("artificial"[All Fields] AND "intelligence"[All Fields]) OR "artificial intelligence"[All Fields]) OR ("neural networks, computer"[MeSH Terms] OR ("neural"[All Fields] AND "networks"[All Fields] AND "computer"[All Fields]) OR "computer neural networks"[All Fields] OR ("neural"[All Fields] AND "network"[All Fields]) OR "neural network"[All Fields]) OR "automat*"[All Fields]
3. #3 "sound"[MeSH Terms] OR "sound"[All Fields] OR "sounded"[All Fields] OR "soundings"[All Fields] OR "sounds"[All Fields] OR "sound s"[All Fields] OR "sounding"[All Fields] OR "speech"[MeSH Terms] OR "speech"[All Fields] OR "speeches"[All Fields] OR "voice"[MeSH Terms] OR "voice"[All Fields] OR "voices"[All Fields] OR "voice s"[All Fields] OR "voiced"[All Fields] OR "voicing"[All Fields] OR "acoustic*"[All Fields] OR "audio"[All Fields] OR "audios"[All Fields] OR "vowel"[All Fields] OR "vowel s"[All Fields] OR "voweled"[All Fields] OR "vowels"[All Fields] OR "vocal"[All Fields] OR "vocales"[All Fields] OR "vocalic"[All Fields] OR "vocalisation"[All Fields] OR "vocalisations"[All Fields] OR "vocalised"[All Fields] OR "vocalising"[All Fields] OR "vocalization"[All Fields] OR "vocalizations"[All Fields] OR "vocalize"[All Fields] OR "vocalized"[All Fields] OR "vocalizer"[All Fields] OR "vocalizers"[All Fields] OR "vocalizes"[All Fields] OR "vocalizing"[All Fields] OR "vocally"[All Fields] OR "vocals"[All Fields] OR "pitch"[All Fields] OR "pitched"[All Fields] OR "pitches"[All Fields] OR "pitching"[All Fields] OR "prosodies"[All Fields] OR "prosody"[All Fields]
4. ((#1) AND (#2)) AND (#3)

**Web of Science Core Collection**

1. (ALL=(depressi*)) OR ALL=(depressive disorder*)
2. ((((ALL=(deep learning)) OR ALL=(machine learning)) OR ALL=(Artificial Intelligence)) OR ALL=(neural network)) OR ALL=(automat*)
3. ((((((((ALL=(sound)) OR ALL=(speech)) OR ALL=(voice)) OR ALL=(acoustic*)) OR ALL=(audio)) OR ALL=(vowel)) OR ALL=(vocal)) OR ALL=(pitch)) OR ALL=(prosody)
4. #3 AND #2 AND #1

**Scopus**

TITLE-ABS-KEY (“depressi*” OR "depressive disorder*”) AND TITLE-ABS-KEY("deep learning" OR "machine learning" OR "Artificial Intelligence" OR "neural network" OR "automat*") AND TITLE-ABS-KEY("sound" OR "speech" OR "voice" OR "acoustic*" OR "audio" OR "vowel" OR "vocal" OR "pitch" OR "prosody" )

**IEEE**

("All Metadata": "depressi*" OR "All Metadata": "depressive disorder*") AND ("All Metadata": "deep learning" OR "All Metadata": "machine learning" OR "All Metadata": "Artificial Intelligence" OR "All Metadata": "neural network" OR "All Metadata": "automat*") AND ("All Metadata": "sound" OR "All Metadata": "speech" OR "All Metadata": "voice" OR "All Metadata": "acoustic*" OR "All Metadata": "audio" OR "All Metadata": "vowel" OR "All Metadata": "vocal" OR "All Metadata": "pitch" OR "All Metadata": "prosody")
