# Supplementary figures and images for: Diagnostic accuracy of deep learning using speech samples in depression: a systematic review and meta-analysis
Source: J Am Med Inform Assoc. 2024 Jul 16;31(10):2394–404. doi: 10.1093/jamia/ocae189 (PMC11413444; doi:10.1093/jamia/ocae189)

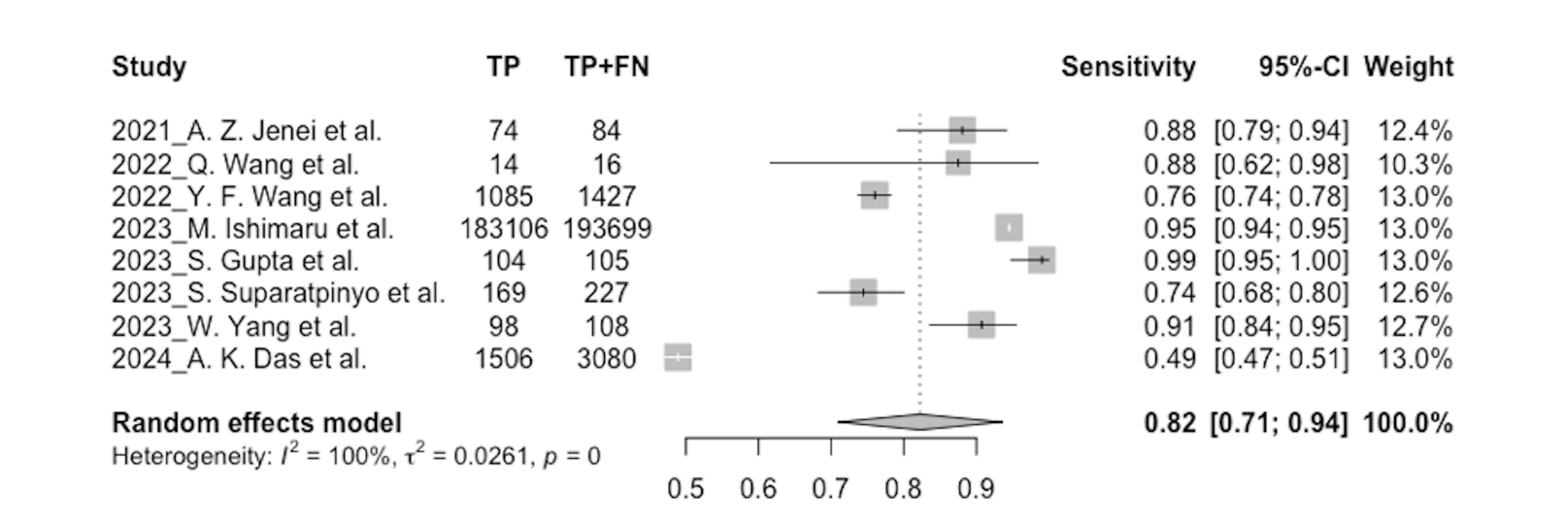

Supplement: ocae189_Supplementary_Data [file ocae189_supplementary_data.zip › ocae189_Supplementary_Data/SF1 Forest plot for the pooled sensitivity.png]

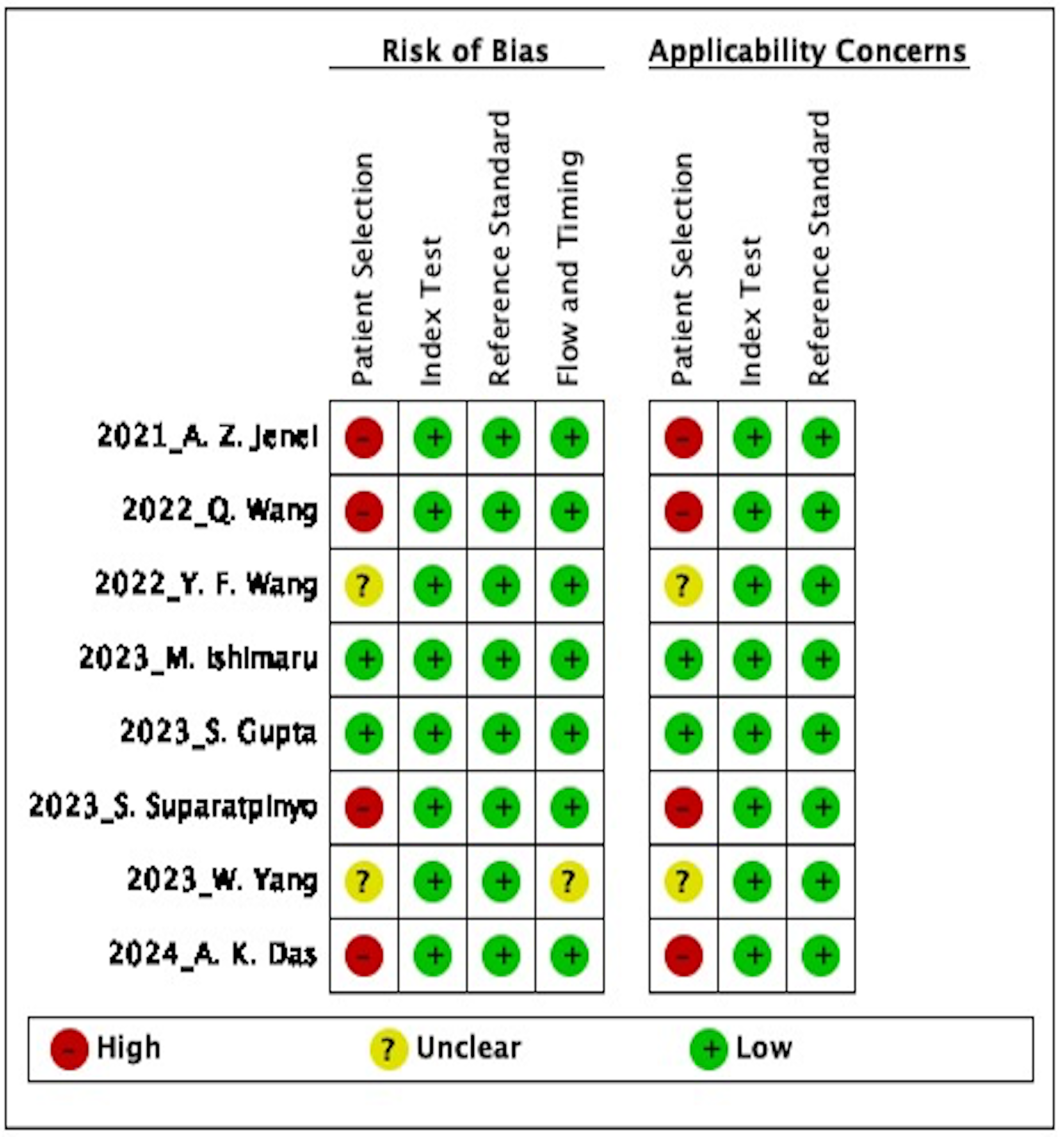

Supplement: ocae189_Supplementary_Data [file ocae189_supplementary_data.zip › ocae189_Supplementary_Data/SF10 Risk of bias and applicability concerns summary.png]

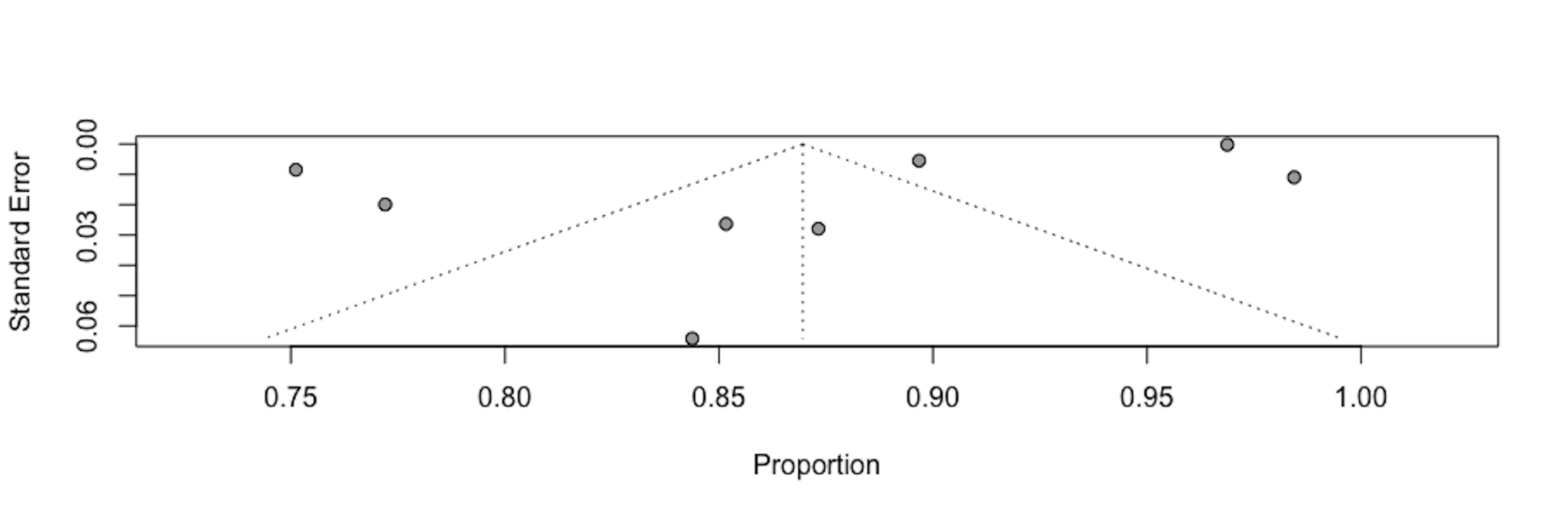

Supplement: ocae189_Supplementary_Data [file ocae189_supplementary_data.zip › ocae189_Supplementary_Data/SF11 Funnel plot for the pooled accuracy.png]

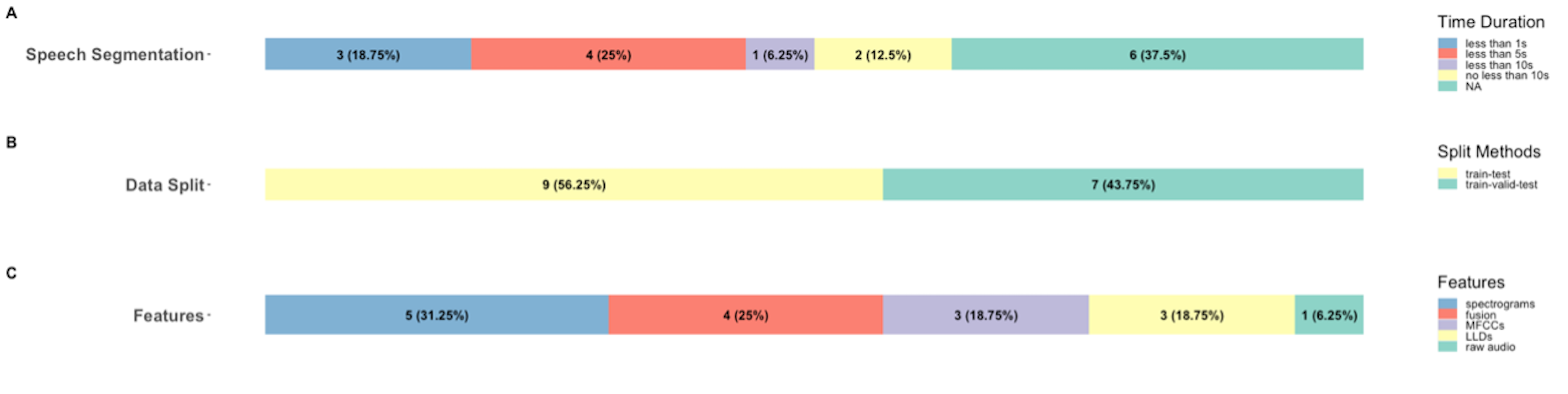

Supplement: ocae189_Supplementary_Data [file ocae189_supplementary_data.zip › ocae189_Supplementary_Data/SF12 Speech processing choices.png]

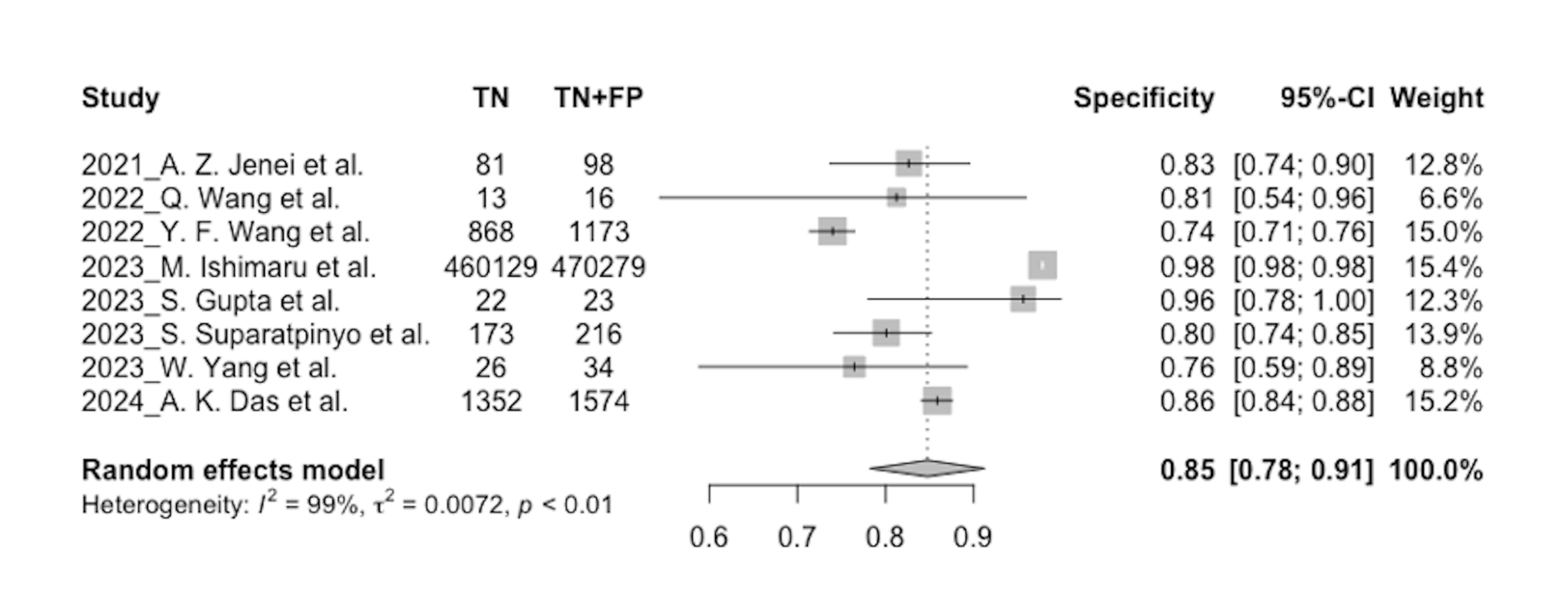

Supplement: ocae189_Supplementary_Data [file ocae189_supplementary_data.zip › ocae189_Supplementary_Data/SF2 Forest plot for the pooled specificity.png]

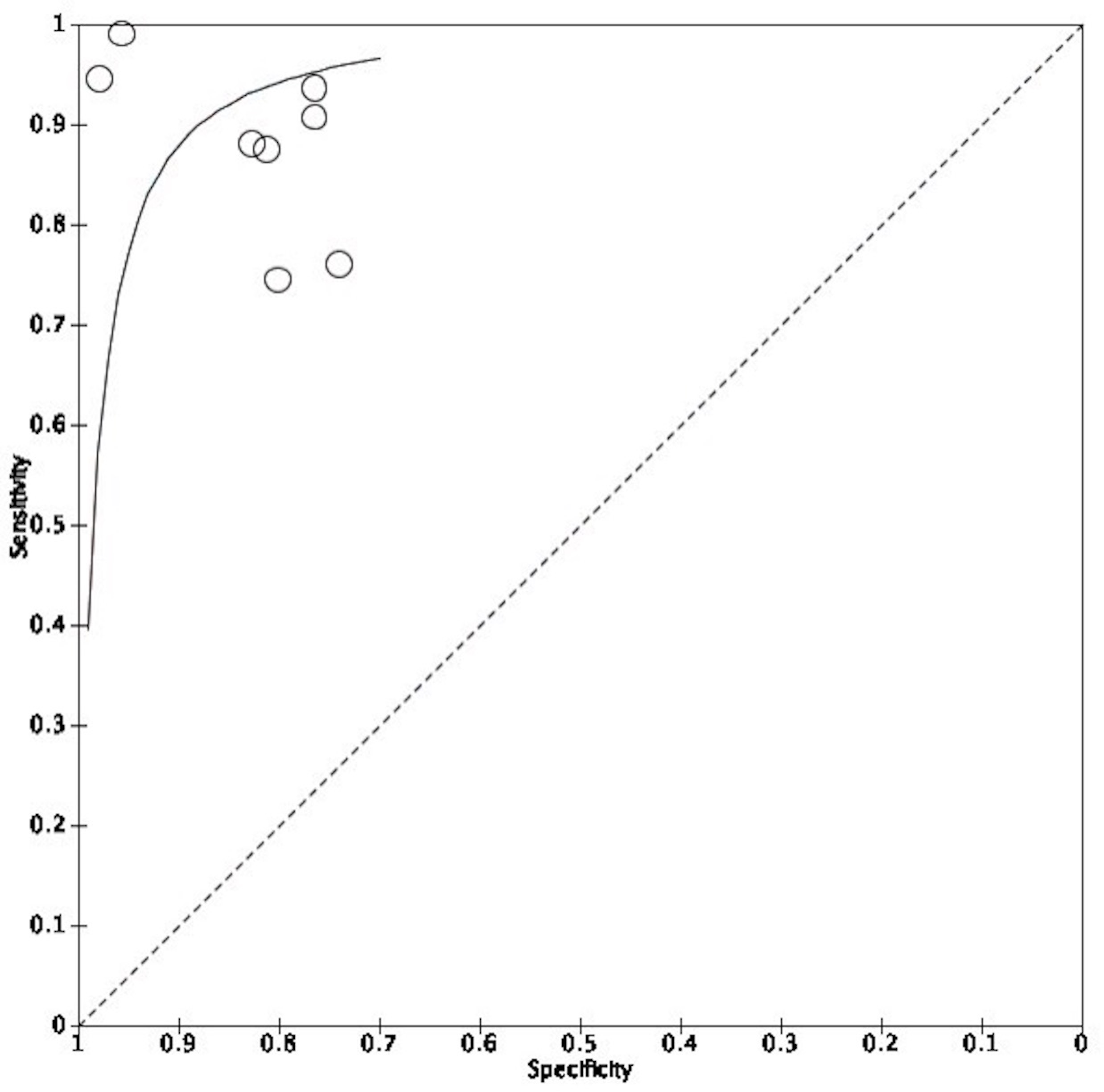

Supplement: ocae189_Supplementary_Data [file ocae189_supplementary_data.zip › ocae189_Supplementary_Data/SF3 SROC curve for the pooled estimate.jpg]

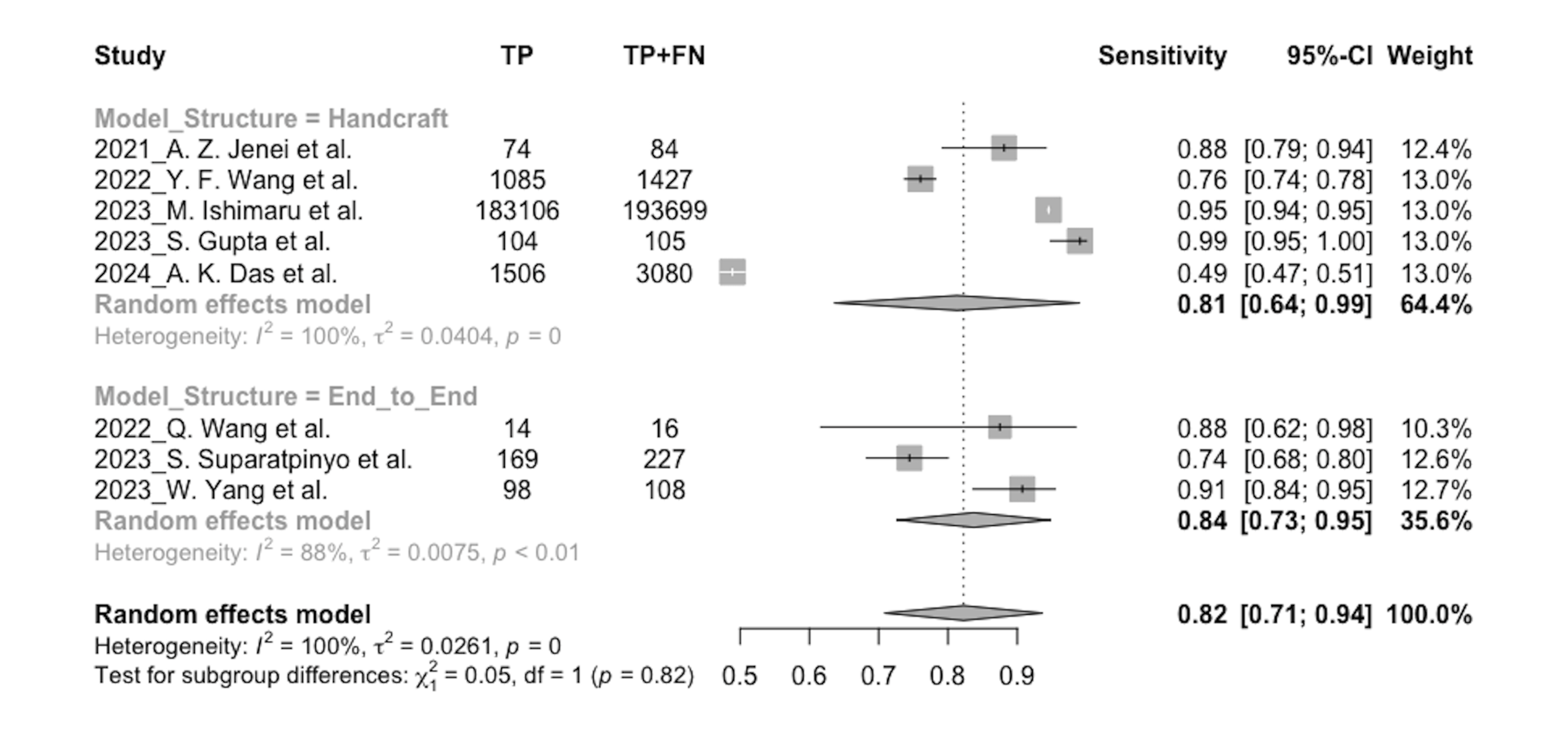

Supplement: ocae189_Supplementary_Data [file ocae189_supplementary_data.zip › ocae189_Supplementary_Data/SF4 Forest plot for the subgroup sensitivity.png]

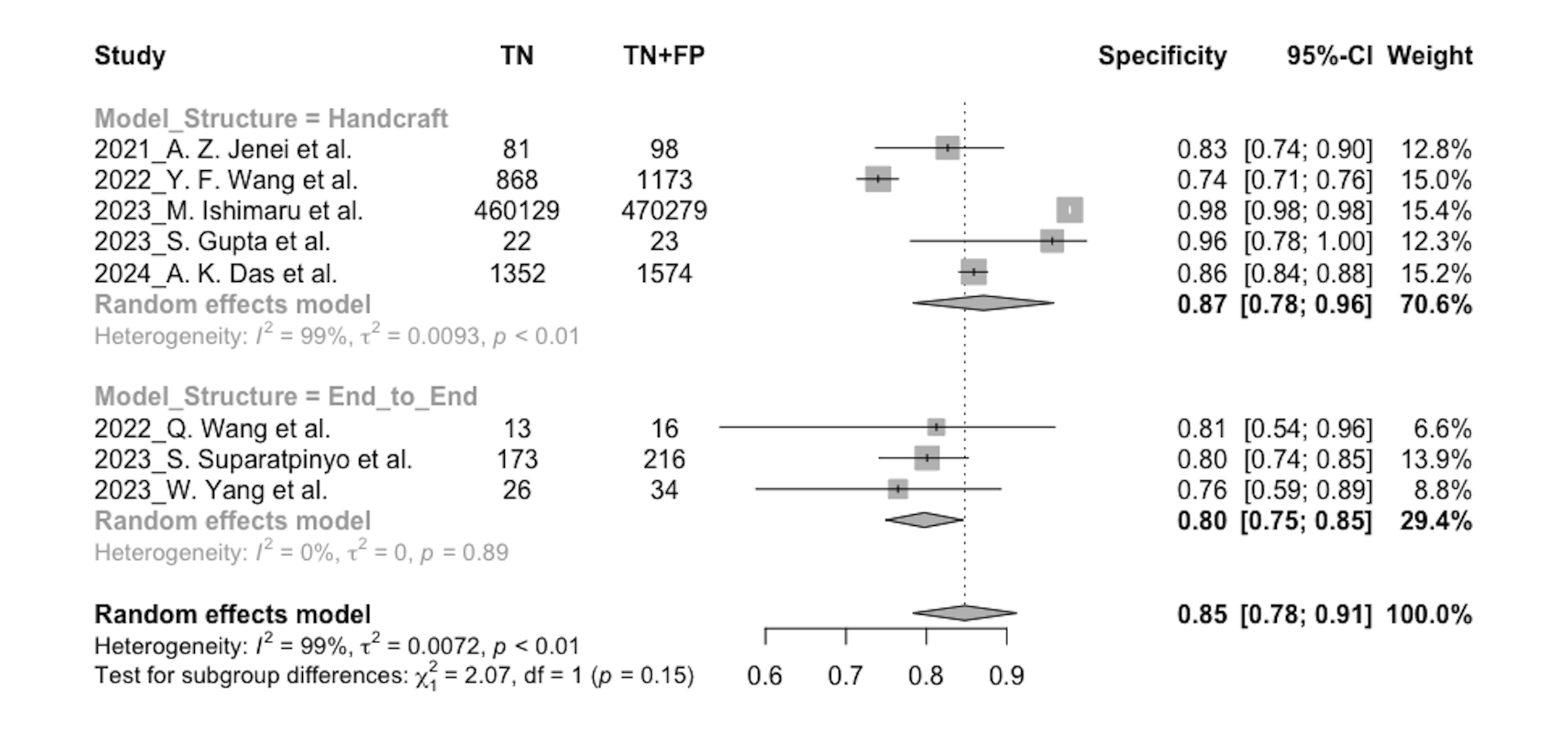

Supplement: ocae189_Supplementary_Data [file ocae189_supplementary_data.zip › ocae189_Supplementary_Data/SF5 Forest plot for the subgroup specificity.png]

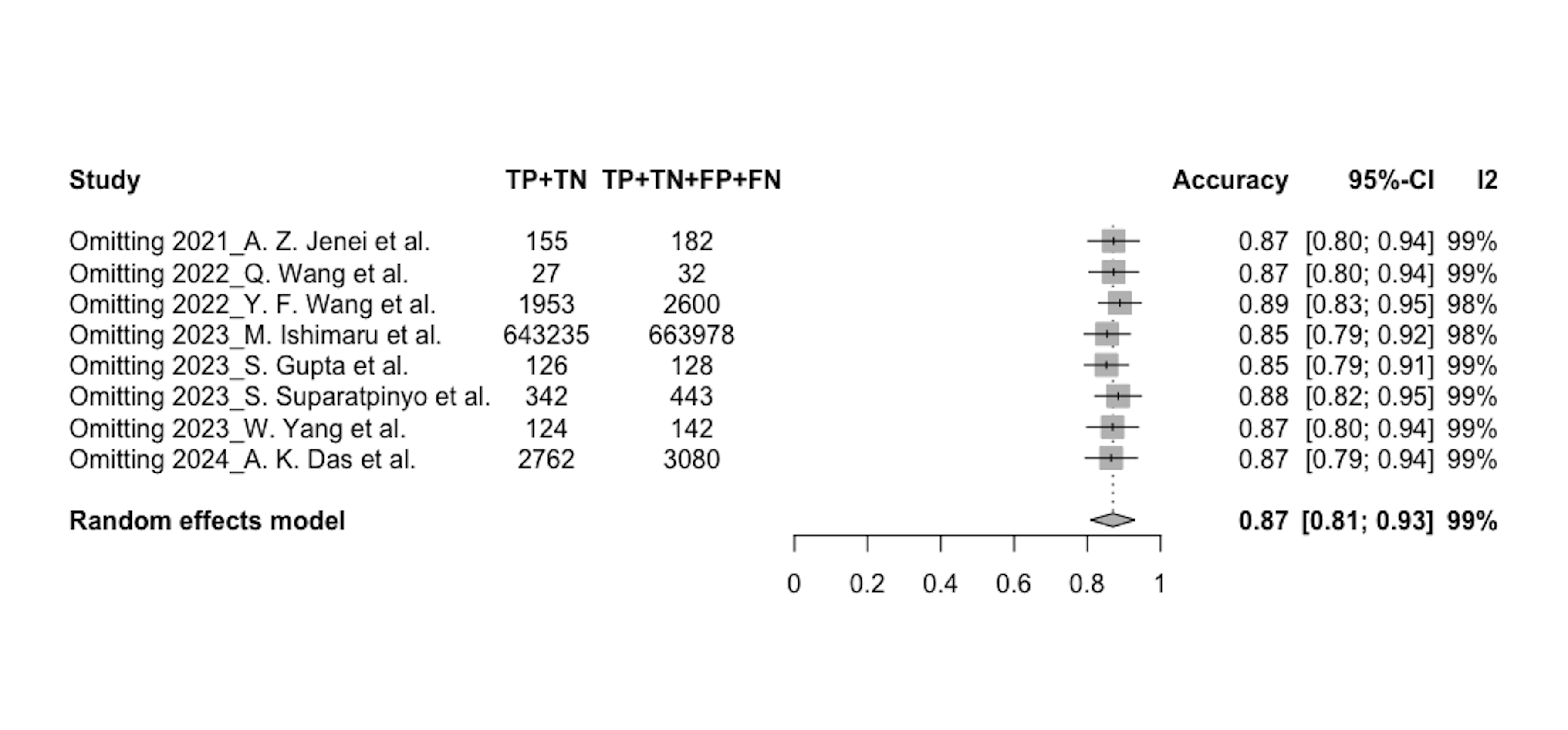

Supplement: ocae189_Supplementary_Data [file ocae189_supplementary_data.zip › ocae189_Supplementary_Data/SF6 Leave one out plot for accuracy.png]

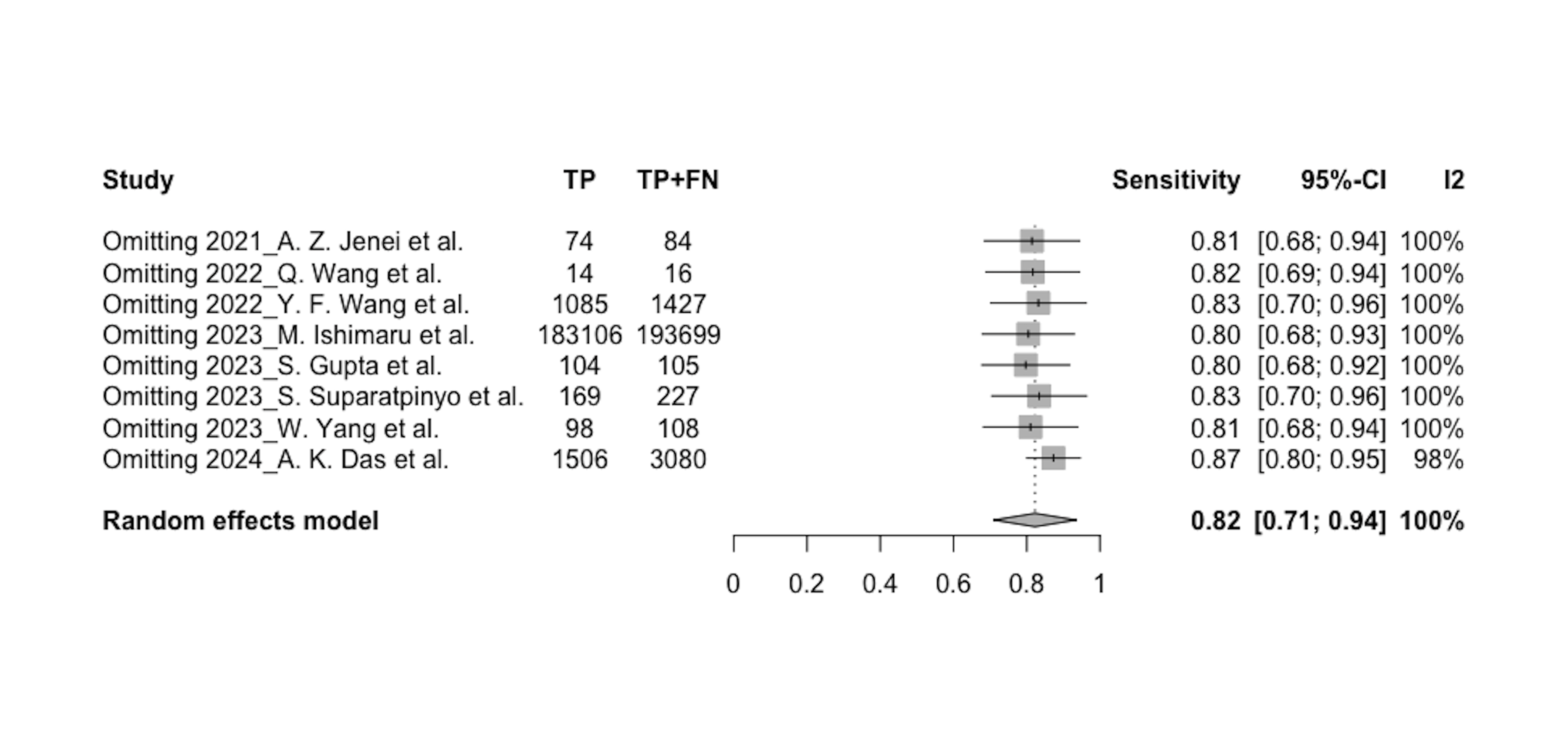

Supplement: ocae189_Supplementary_Data [file ocae189_supplementary_data.zip › ocae189_Supplementary_Data/SF7 Leave one out plot for sensitivity.png]

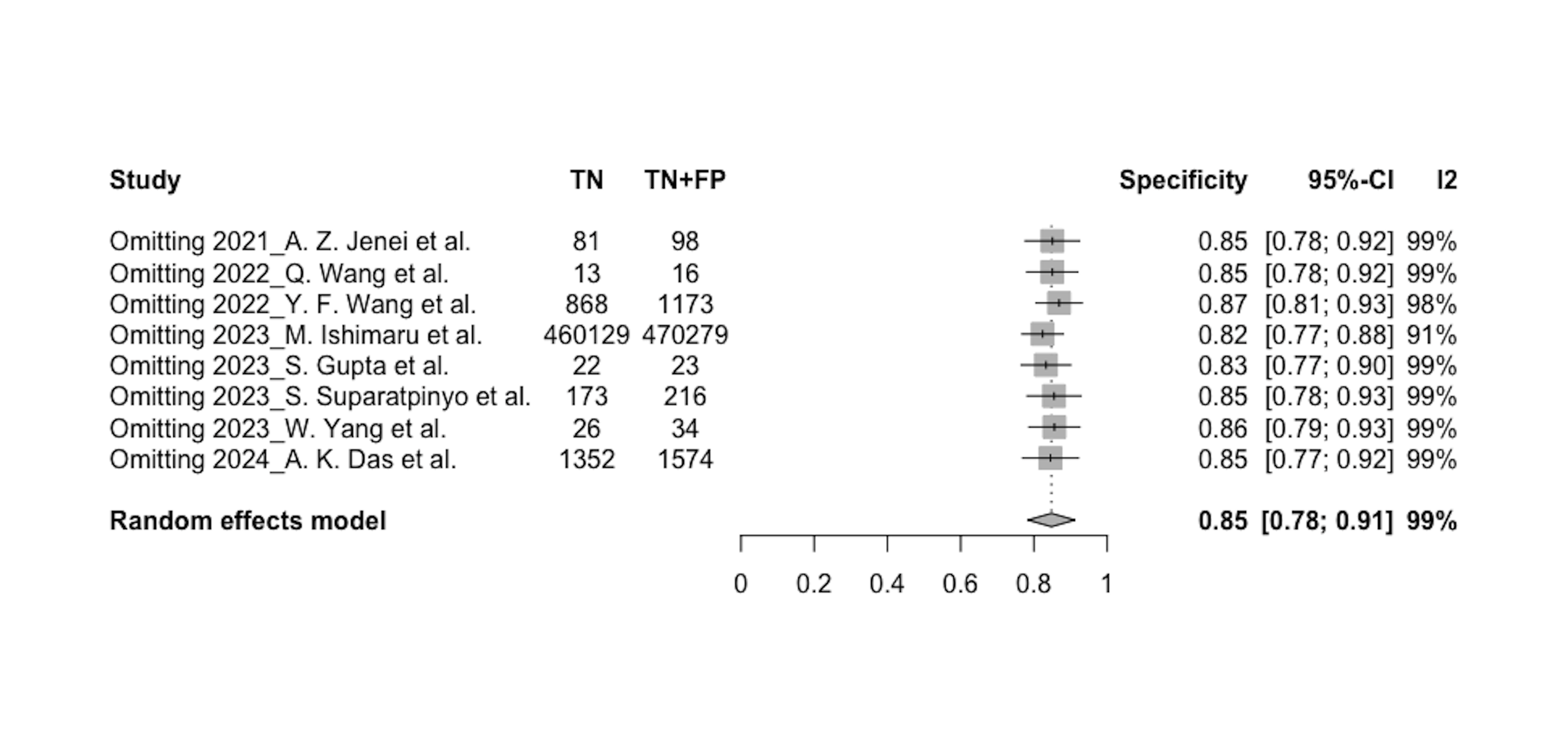

Supplement: ocae189_Supplementary_Data [file ocae189_supplementary_data.zip › ocae189_Supplementary_Data/SF8 Leave one out plot for specificity.png]

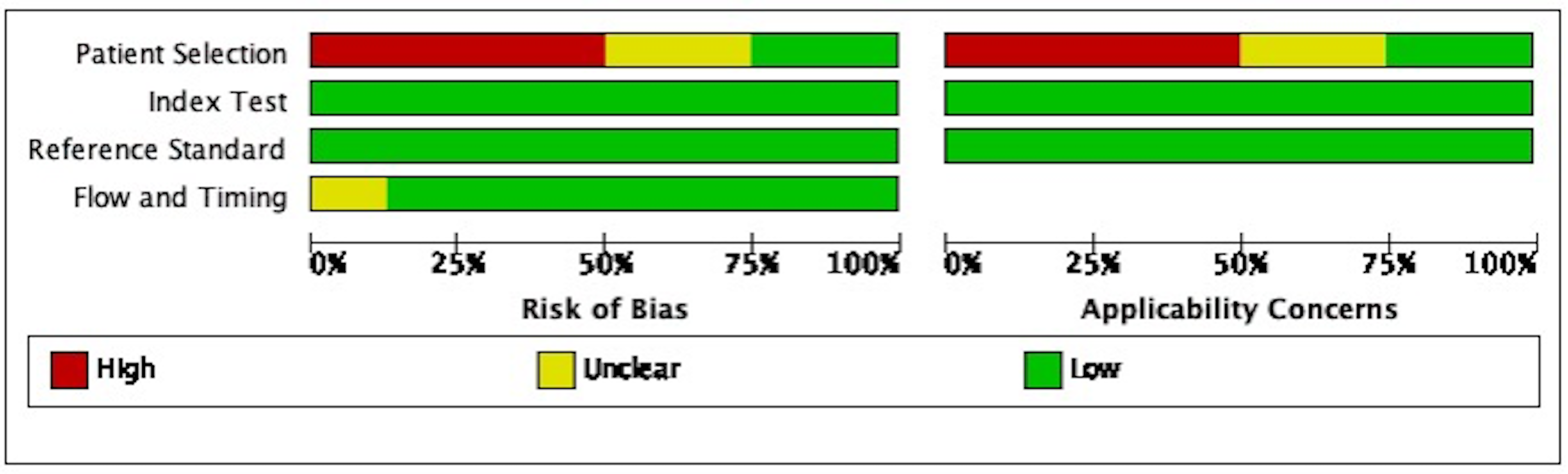

Supplement: ocae189_Supplementary_Data [file ocae189_supplementary_data.zip › ocae189_Supplementary_Data/SF9 Risk of bias and applicability concerns graph.png]
